# Dual defense: melatonin simultaneously mitigates cadmium toxicity and southern blight in peanut

**DOI:** 10.3389/fpls.2025.1686151

**Published:** 2025-10-16

**Authors:** Suling Sang, Shan Tian, Wen Xu, Haojie Shi, Benliang Deng

**Affiliations:** ^1^ Institute of Plant Protection, Henan Academy of Agricultural Sciences, Zhengzhou, Henan, China; ^2^ Life Science College, Luoyang Normal University, Luoyang, Henan, China; ^3^ College of Advanced Agricultural Sciences, Zhejiang A&F University, Hangzhou, Zhejiang, China

**Keywords:** melatonin, cadmium stress, disease resistance, metabolic reprogramming, peanut

## Abstract

Peanut (*Arachis hypogaea* L.), a vital oilseed crop, faces severe yield losses from Southern blight (*Sclerotium rolfsii*) and cadmium (Cd) contamination in agricultural soils. While crops often endure combined abiotic-biotic stresses in nature, synergistic solutions remain limited. Here, we investigated melatonin’s dual role in enhancing Cd detoxification and disease resistance using physiological assays and LC-MS metabolomics in peanut seedlings. Under combined stress (1 mM CdCl_2_ + *S. rolfsii*), melatonin treatment reduced disease incidence by 75% and Cd accumulation by 57% (roots) and 37% (shoots). Mechanistically, melatonin upregulated jasmonic acid biosynthesis genes (*AhLOX7, AhOPR3*) and ABC transporter genes (*ABCC3, ABCC4*), potentiated the antioxidant system (SOD: +135%; CAT: +128%), and significantly mitigated Cd-induced oxidative damage. Metabolomics revealed preferential resource allocation to defense metabolites, with marked accumulation of key antioxidants (e.g., uric acid) and defense phytohormones (e.g., salicylic acid, jasmonic acid) over primary metabolism. These findings demonstrate melatonin’s capacity to rewire stress-responsive metabolism, balancing Cd detoxification and pathogen defense without growth penalties. Our study provides the first evidence that melatonin confers dual resistance by reprogramming defense-related pathways, offering a scalable strategy for cultivating stress-resilient peanuts in contaminated agroecosystems, with implications for similar oilseed crops.

## 1 Introduction

As a pivotal oilseed crop contributing ~20% of China’s edible oil output (Zhao et al., 2021), peanut (*Arachis hypogaea* L.) is also a key industrial resource for biodiesel and food additives (Hariharan et al., 2021). Yet, its productivity is severely threatened by Southern blight (SB; *Sclerotium rolfsii*), which causes up to 50% yield losses in peanuts through *sclerotia*-mediated root/stem maceration (Marinelli et al., 1998; Shew and Henn, 2022). This soil-borne pathogen persists for years, with post-harvest conditions accelerating its proliferation. Compounding this, cadmium (Cd) contaminates 19.4% of China’s farmland (Zou et al., 2021), accumulating in peanut kernels at levels exceeding the safety threshold for edible oils (Shi et al., 2014; Wang et al., 2015).

Peanuts may suffer from simultaneous Cd toxicity and *Sclerotium rolfsii* infection, severely reducing yield and quality (Shi et al., 2014; Ozgonen et al., 2010). Although combined treatments (e.g., nanoparticles + microbes) have been reported to alleviate heavy metal and disease stresses (Khan et al., 2023), no single substance has yet been shown to mitigate both concurrently (Pandey et al., 2017). This gap underscores the need for innovative solutions to address co-occurring abiotic and biotic stresses in crops (Dixit et al., 2024).

Cd-contaminated soil significantly reduces peanut yield (e.g., biomass decline by 20–40%) and seed quality (e.g., increased Cd accumulation up to 0.5–1.2 mg/kg), as demonstrated in field trials by Shi et al. (2014). As a redox-active metal, Cd disrupts cellular homeostasis, compromising both crop quantity and oil quality (Nouairi et al., 2005). Although plants deploy antioxidants (Gu et al., 2021), ROS paradoxically increase SB susceptibility (Morkunas et al., 2018), creating an urgent need for integrated solutions (Kissoudis et al., 2014).

Melatonin, a phylogenetically conserved phytoprotectant with proven scalability in field applications (Kaya and Doganlar, 2019; Tiwari et al., 2020), addresses this paradox. For example, melatonin enabled pepper plants to concurrently resist drought and bacterial infection by enhancing antioxidant capacity, priming defense genes, and harmonizing cross-talk between abiotic/biotic stress pathways (Kaya and Doganlar, 2019). Beyond ROS scavenging (Gu et al., 2021), melatonin differentially regulates Cd uptake: in non-accumulators like rice, it reduces absorption primarily through enhancing cell wall fixation and nitric oxide-mediated transporter regulation, with auxiliary contributions from vacuolar sequestration (Huang et al., 2023), a trait exploitable for cleaner crop production. Its dual role in metal detoxification and pathogen defense aligns with industrial needs for multi-stress-tolerant cultivars. However, its impact on necrotroph resistance under Cd stress remains unclear—a knowledge gap with direct implications for peanut processing industries.

We hypothesized that melatonin primes peanut seedlings for enhanced dual stress resistance by coordinately modulating defense pathways and Cd detoxification. To test this, we aimed to: (A) explore how melatonin affects peanut resistance to *S. rolfsii* under Cd stress, (B) elucidate its physiological mechanisms, and (C) identify key metabolic shifts via untargeted metabolomics. Using combined Cd and pathogen challenge, we assessed disease incidence, antioxidant responses, and Cd accumulation, complemented by LC-MS to decode metabolic reconfiguration. Our findings reveal melatonin’s role in balancing Cd exclusion and pathogen defense, offering a scalable solution for contaminated agroecosystems.

## 2 Materials and methods

### 2.1 Experimental design

The study employed ‘Yuhua 22’ (*Arachis hypogaea* L.), a Southern blight (SB)-susceptible peanut cultivar (Fan et al., 2024). Uniform seeds (5.0-6.5 mm diameter, ≥95% viability) were surface-sterilized with 1% NaOCl for 5 min followed by thorough rinsing. Seedlings were grown in vermiculite under controlled greenhouse conditions (28-30°C, 65 ± 2% humidity, 12-h photoperiod) with half-strength Hoagland solution irrigation.

The *S. rolfsii* inoculum (strain HN2024-01, isolated from infected peanut plants in Henan Province; Fan et al., 2024) was prepared by growing the fungus on Potato Dextrose Agar (PDA) plates at 28°C for 5 days in the dark. Mycelial plugs (5 mm diameter) were excised from the actively growing margin of the colony. The mycelial plug method was selected for its proven efficacy and reliability in establishing a robust and consistent infection under controlled conditions, as it delivers a high density of viable mycelia directly to the host tissue. At the two-week growth stage, plants were inoculated with *S. rolfsii* by carefully placing five mycelial plugs in direct contact with the stem base and surrounding soil near the root-stem junction per plant. This quantity was determined based on preliminary pathogenicity tests to ensure the development of clear disease symptoms in the susceptible cultivar under our experimental conditions.

The concentration of melatonin (0.1 mM) used for foliar application was selected based on preliminary dose-response experiments. Peanut seedlings under combined SB+Cd stress were treated with a range of MT concentrations (0.01, 0.05, 0.1, 0.5, and 1.0 mM). Key endpoints, including disease incidence (%), Cd accumulation in shoots (μg/g DW), and seedling biomass (g/plant), were evaluated. The 0.1 mM concentration was identified as optimal, providing the most significant reduction in disease severity and Cd accumulation while promoting growth. Lower concentrations (e.g., 0.01 mM) showed negligible effects, while higher concentrations (e.g., 1.0 mM) exhibited inhibitory effects on plant growth even in the absence of stress (data presented in **Supplementary Table S1**).

Eight treatments (five replicates each) were established: (1) Control (no stress), (2) Melatonin only (MT, 0.1 mM), (3) Cd stress (1 mM CdCl_2_), (4) Cd + MT, (5) Pathogen only (*S. rolfsii*, SB), (6) Pathogen + melatonin (SB+MT), (7) Pathogen + Cd (SB+Cd), and (8) Pathogen + Cd + melatonin (SB+Cd+MT). CdCl_2_ was applied through root irrigation, while melatonin was foliar-sprayed (10 mL/plant/day for 3 consecutive days).

Seven days post-treatment, roots and shoots were collected for Cd content analysis (atomic absorption spectroscopy after acid digestion). Stem samples were immediately frozen in liquid nitrogen for subsequent physiological analyses, including H_2_O_2_ and TBARS quantification, defense enzyme activity assays, and phenolic and free amino acid content determination. For non-targeted metabolomic profiling, stem tissues were specifically sampled only from the dual-stress group (SB+Cd, Jing_B; control) and triple-treatment group (SB+Cd+MT, Jing_A; treatment group).

### 2.2 Physiological parameter analysis and data processing

Oxidative stress markers were quantified using established methods: H_2_O_2_ content was measured via the FOX assay (Gay et al., 1999), where tissue extracts in acetone reacted with xylenol orange/ferrous sulfate, with absorbance read at 560 nm. Lipid peroxidation (TBARS) was determined by heating trichloroacetic acid-homogenized samples with thiobarbituric acid and measuring absorbance at 532 nm (Heath and Packer, 1968). Free amino acids were assessed through ninhydrin reaction at 570 nm (Yemm and Cocking, 1955), while Cd content was analyzed via flame atomic absorption spectrometry after acid digestion (Shi et al., 2014).

Soluble protein was quantified by the Bradford method (Bradford, 1976). For enzyme extraction, frozen stem tissues (0.5 g) were homogenized in 5 mL of ice-cold 50 mM sodium phosphate buffer (pH 7.0) containing 1% (w/v) polyvinylpyrrolidone (PVP) and 1 mM EDTA. The homogenate was centrifuged at 10,000 ×g for 20 min at 4°C, and the supernatant was used for enzyme assays. Defense enzyme activities were assayed in phosphate buffer-extracted stem tissues (10,000 ×g supernatant). SOD activity was evaluated by nitroblue tetrazolium inhibition at 560 nm (Giannopolitis and Ries, 1977), using a reaction mixture containing 50 mM phosphate buffer (pH 7.8), 13 mM methionine, 75 μM nitroblue tetrazolium (NBT), 0.1 mM EDTA, 2 μM riboflavin, and 0.1 mL enzyme extract, with one unit causing 50% inhibition. CAT activity was determined via H_2_O_2_ decomposition at 240 nm (ϵ = 39.4 mM^−1^ cm^−1^; Aebi, 1984) using a reaction mixture containing 50 mM phosphate buffer (pH 7.0), 15 mM H_2_O_2_, and 0.1 mL enzyme extract. PPO activity was measured using catechol as substrate in a reaction mixture containing 50 mM phosphate buffer (pH 6.8), 20 mM catechol, and 0.1 mL enzyme extract, with absorbance monitored at 420 nm for 1 min (Mayer and Harel, 1979). PAL activity was determined by measuring cinnamic acid production at 290 nm (Bolwell et al., 1986) using a reaction mixture containing 50 mM Tris-HCl buffer (pH 8.8), 20 mM L-phenylalanine, and 0.2 mL enzyme extract, incubated at 40 °C for 1 h. All enzyme activities were normalized to protein content and expressed as units per mg protein. Non-enzymatic antioxidants were analyzed as follows: Total glutathione (GSH+GSSG) was quantified via enzymatic cycling after 2-vinylpyridine derivatization (Nagalakshmi and Prasad, 2001). Total phenolics extracted in 80% methanol were measured by Folin-Ciocalteu assay at 765 nm (Singleton and Rossi, 1965), calibrated against gallic acid (0–500 mg/L). FRAP assay determined antioxidant capacity using TPTZ-Fe^3+^ reagent at 593 nm (Benzie and Strain, 1996).

Disease resistance was evaluated by inoculating plants with *S. rolfsii*-infected sorghum grains (3 g/plant; Jia et al., 2023). After 5 days, severity was scored (0–4 scale), with disease incidence rate (DIR) and index (DI) calculated as percentages of infected plants and weighted scores, respectively. Statistical analysis employed a completely randomized design with five biological replicates. Data were subjected to Duncan’s multiple range test (P < 0.05) using IBM SPSS Statistics 20 (IBM Corp., USA).

### 2.3 Extraction and assay of salicylic acid, jasmonic acid, and suberic acid

The extraction and purification of salicylic acid (SA), jasmonic acid (JA), and suberic acid from plant tissues were performed using a modified protocol optimized for compatibility with enzyme-linked immunosorbent assay (ELISA). Fresh plant tissue (approximately 0.2 g) was rapidly harvested, immediately frozen in liquid nitrogen, and ground to a fine powder using a pre-chilled mortar and pestle. The homogenized powder was transferred to a 5 mL microcentrifuge tube, and 2 mL of ice-cold extraction buffer (90% methanol containing 1% acetic acid) was added. The mixture was vortexed vigorously for 30 seconds and then incubated at 4°C for 12 hours under continuous shaking at 150 rpm to ensure complete extraction of target compounds. Following incubation, the sample was centrifuged at 12,000 × g for 15 minutes at 4°C, and the supernatant was carefully collected. The pellet was re-extracted with an additional 1 mL of the same extraction buffer, followed by centrifugation and combination of the supernatants. For purification, the combined supernatant was evaporated to complete dryness under a gentle stream of nitrogen gas at 40°C. The dried residue was reconstituted in 0.5 mL of phosphate-buffered saline (PBS, 0.01 M, pH 7.4) by vortexing for 2 minutes. The reconstituted sample was then centrifuged at 12,000 × g for 10 minutes at 4°C to remove any insoluble particles, and the final supernatant was aliquoted and stored at -80°C until ELISA analysis. This method effectively eliminates interfering substances such as pigments and lipids while maintaining the structural integrity and immunoreactivity of SA, JA, and suberic acid, thereby ensuring accurate quantification using specific ELISA kits. The concentrations of SA, JA, and suberic acid were quantified using commercial enzyme-linked immunosorbent assay (ELISA) kits following the manufacturers’ protocols. The kits used were as follows: JA was analyzed with kit No. ml077234, SA with kit No. ml077224 (both from Shanghai Enzyme-linked Biotechnology Co., Ltd., China), and suberic acid with kit No. F0415-OA (FANKEW, Shanghai, China). Briefly, fresh plant samples were homogenized in phosphate buffer (pH 7.4) and centrifuged at 12,000 × g for 15 min at 4°C. The supernatant was collected and used for analysis. Microplates pre-coated with specific antibodies were incubated with sample extracts or standards, followed by the addition of horseradish peroxidase (HRP)-conjugated detection antibodies. After washing, tetramethylbenzidine (TMB) substrate was added for color development, and the absorbance was measured at 450 nm. The concentrations of suberic acid, JA, SA, and uric acid were calculated based on standard curves.

### 2.4 Gene expression analysis by quantitative real-time PCR

Total RNA was extracted from stem tissues of peanut seedlings using the RNAprep Pure Plant Plus Kit (Polysaccharides & Polyphenolics-rich) (Tiangen, China) according to the manufacturer’s instructions. First-strand cDNA was synthesized from 1 µg of DNase I-treated total RNA using the PrimeScript RT reagent Kit with gDNA Eraser (Takara, Japan). Quantitative real-time PCR was performed on a QuantStudio 5 Real-Time PCR System (Applied Biosystems, USA) using TB Green Premix Ex Taq II (Takiara, Japan). The 20 µL reaction mixture contained 10 µL of TB Green Premix, 0.8 µL of each gene-specific primer (10 µM), 2 µL of diluted cDNA, and 6.4 µL of nuclease-free water. The expression levels of four target genes, *AhLOX7*, *AhOPR3*, *ABCC3*, and *ABCC4*, were analyzed. The amplification program was as follows: initial denaturation at 95°C for 30 s, followed by 40 cycles of 95 °C for 5 s and 60°C for 34 s. A melt curve analysis was conducted at the end of each run to confirm the specificity of amplification. The relative gene expression levels were calculated using the 2−ΔΔCT method, with *β-actin* serving as the internal reference gene. All gene-specific primer sequences used in this study are listed in **Supplementary Table S2**.

### 2.5 Metabolite extraction and untargeted metabolomics analysis

For metabolite extraction, approximately 80 mg of peanut stem tissue was flash-frozen in liquid nitrogen and ground to a fine powder using a mortar and pestle. The powdered tissue was extracted with 1000 μL of methanol/acetonitrile/water (2:2:1, v/v/v) following the protocol of De Vos et al. (2007). After centrifugation at 14,000 g for 20 min at 4°C, the supernatant was vacuum-dried and reconstituted in 100 μL of acetonitrile/water (1:1, v/v). The solution was re-centrifuged (14,000 g, 4°C, 15 min), and the clarified supernatant was directly injected for LC-MS analysis.

Metabolite profiling was performed using an Agilent 1290 Infinity UHPLC system coupled to a SCIEX TripleTOF 6600 mass spectrometer (Zhang et al., 2022). Chromatographic separation was achieved on an ACQUITY UPLC BEH Amide column (2.1 × 100 mm, 1.7 μm) with mobile phase A (25 mM ammonium acetate/ammonium hydroxide in water) and mobile phase B (acetonitrile). The gradient program was as follows: 95% B (0.5 min), decreased to 65% B (6.5 min), then to 40% B (1 min), held for 1 min, and returned to 95% B (0.1 min) with 3 min re-equilibration. Electrospray ionization (ESI) conditions were: Gas1/Gas2 at 60 psi, curtain gas (CUR) at 30 psi, source temperature at 600 °C, and ion spray voltage floating (ISVF) at ±5500 V. Mass spectrometry analysis included full-scan MS (60–1000 m/z, 0.20 s/spectrum) and auto-MS/MS (25–1000 m/z, 0.05 s/spectrum) in information-dependent acquisition (IDA) mode. Key parameters were: collision energy (CE) = 35 ± 15 eV, declustering potential (DP) = ± 60 V, with exclusion of 4 Da isotopes and selection of 10 candidate ions per cycle.

### 2.6 Data processing and statistical analysis

Raw MS data were converted to MzXML format using ProteoWizard MSConvert (Chambers et al., 2012) and processed with XCMS (Tautenhahn et al., 2008) for peak detection (centWave algorithm: Δm/z 10 ppm, peakwidth = c(10, 60), prefilter = c(10, 100)) and peak grouping (bw = 5, mzwid = 0.025, minfrac = 0.5). Isotope and adduct annotation was performed using CAMERA (Kuhl et al., 2012), followed by feature filtering (retaining features with >50% non-zero values in at least one group) (Parsons et al., 2007). Metabolite identification was conducted with a tiered confidence level approach (Summer et al., 2007). Level 1 identification (confidently identified compounds) was achieved by matching both the accurate mass (< 10 ppm error) and the MS/MS fragmentation spectra against our in-house database built with authentic standards. The in-house database comprises over 500 standard compounds, primarily covering key classes of primary (e.g., organic acids, amino acids, sugars) and secondary (e.g., phenolics, flavonoids, alkaloids, terpenoids) metabolites relevant to plant stress responses. The MS/MS matching was performed with a fragment ion tolerance of 0.02 Da. For compounds without an MS/MS match in the database, Level 2 identification (putatively annotated compounds) was assigned based on accurate mass matching (< 10 ppm) against online databases (e.g., HMDB, KEGG).

Sum-normalized data were analyzed using multivariate statistics (ropls package). Pareto-scaled PCA and OPLS-DA were conducted, with model validation via 7-fold cross-validation and permutation testing (Thévenot et al., 2015). Metabolite significance was determined by VIP > 1 (group discrimination), *p* < 0.05 (statistical significance, assessed by Student’s t-test), and FC >1.5 (biological relevance) (Xia et al., 2015; Xia and Wishart, 2016). Variable correlations were assessed using Pearson’s method (Patti et al., 2012).

## 3 Results

### 3.1 Optimizing melatonin concentration enhances peanut seedling resilience under dual stress

To determine the optimal concentration of melatonin for alleviating combined cadmium (Cd) and southern blight stress in peanut seedlings, we evaluated the effects of different melatonin concentrations (0, 0.01, 0.1, and 1.0 mM) on dry weight, disease index, and Cd accumulation in the plants after 7 days of treatment. As summarized in **Supplementary Table S1**, melatonin application at 0.1 mM significantly improved plant growth under dual-stress conditions, resulting in the highest dry weight (2.43 g seedling^-1^) among all treatments. In contrast, the lowest melatonin concentration (0.01 mM) showed only marginal improvements in dry weight (2.39 g seedling^-1^) and moderate mitigation of stress symptoms. Notably, the highest melatonin dose (1.0 mM) led to a pronounced reduction in dry weight (2.15 g seedling^-1^), suggesting a phytotoxic effect when applied at excessive levels.

Similarly, the disease index, reflecting the severity of southern blight infection, was most effectively reduced by the 0.1 mM melatonin treatment (8.9%), demonstrating a strong protective effect against pathogen progression. While the 0.01 mM treatment also offered some protection (19.3%), it was significantly less effective. Conversely, 1.0 mM melatonin, despite reducing disease index relative to the control (12.5%), was less effective than the 0.1 mM treatment and coincided with observed growth inhibition.

Furthermore, melatonin at 0.1 mM markedly decreased Cd accumulation in plant tissues (114.7 mg kg^-1^ DW), underscoring its role in enhancing heavy metal detoxification. The 0.01 mM treatment moderately reduced Cd content (205.8 mg kg^-1^ DW), whereas the 1.0 mM concentration resulted in a higher Cd accumulation (145.7 mg kg^-1^ DW) compared to the 0.1 mM group, further supporting its suboptimal performance and potential toxicity.

Collectively, these results indicate that 0.1 mM melatonin provides the most effective enhancement of plant resilience under combined Cd and biotic stress, significantly improving biomass, reducing disease symptoms, and mitigating Cd uptake. Lower concentrations (0.01 mM) offered limited benefits, and higher concentrations (1.0 mM) induced adverse effects, thereby justifying the selection of 0.1 mM melatonin for subsequent experiments.

### 3.2 Disease incidence, oxidative stress markers, and cadmium accumulation

**Figure 1:**
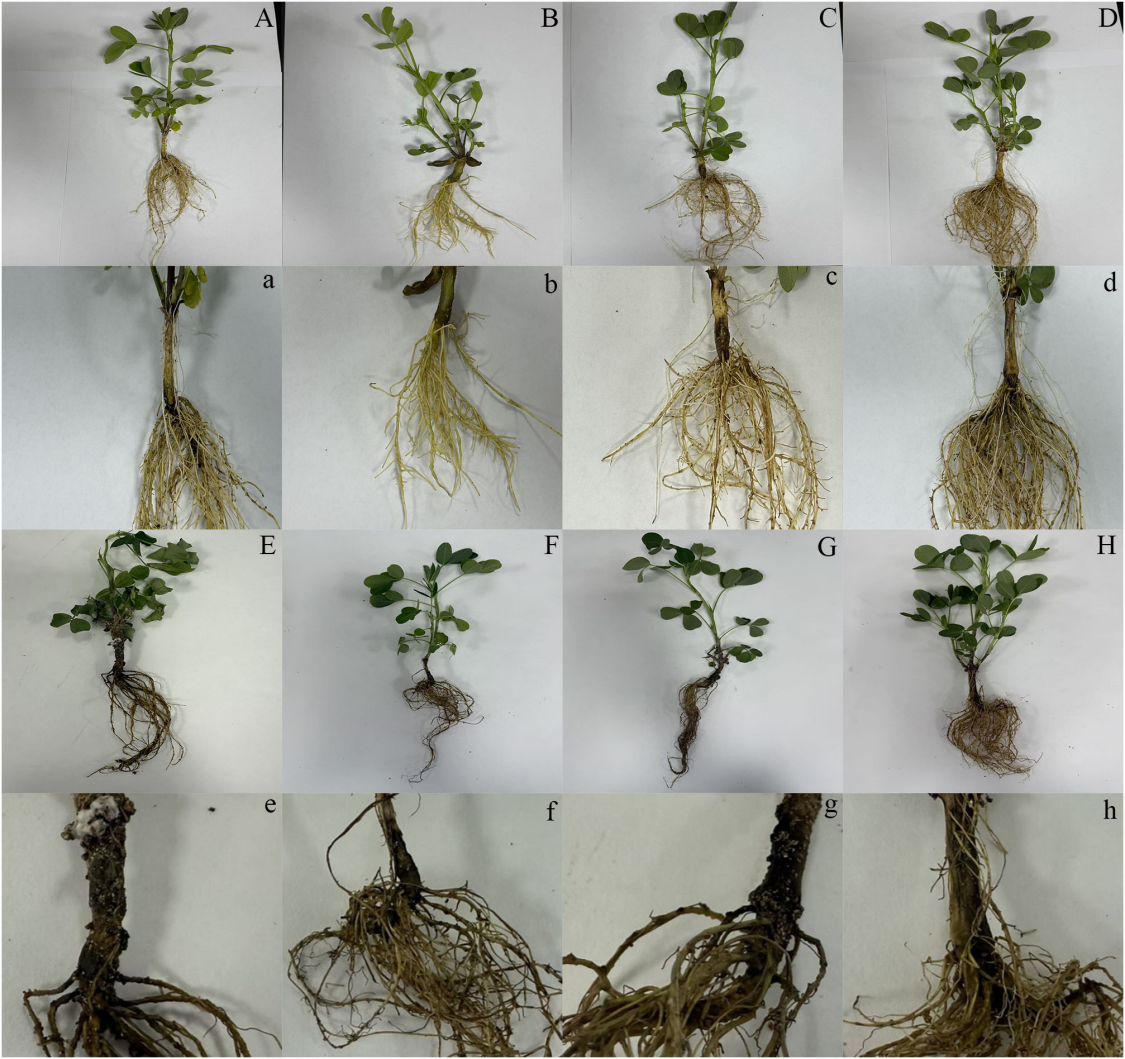
Effects of different treatments on disease development in peanut seedlings. Phenotypic variation of 7-day-old peanut seedlings at 7 days post-inoculation with *Sclerotium rolfsii* (n ≥ 5 plants per treatment). From left to right: **(A, a)** Water control (mock-treated plants); **(B, b)** Melatonin (MT) alone (0.1 mM foliar spray for 3 consecutive days); **(C, c)** Cadmium **(C, d)** alone (1 mM CdCl_2_ soil irrigation); **(D, d)** Cd + MT; **(E, e)** Pathogen alone (*S. rolfsii* inoculation at the root–stem junction); **(F, f)** Pathogen + MT; **(G, g)** Pathogen + Cd; **(H, h)** Pathogen + Cd + MT. Plants were grown under controlled conditions: 28–30 °C, 65% relative humidity, and a 12-h light/12-h dark photoperiod.

**Figure 2:**
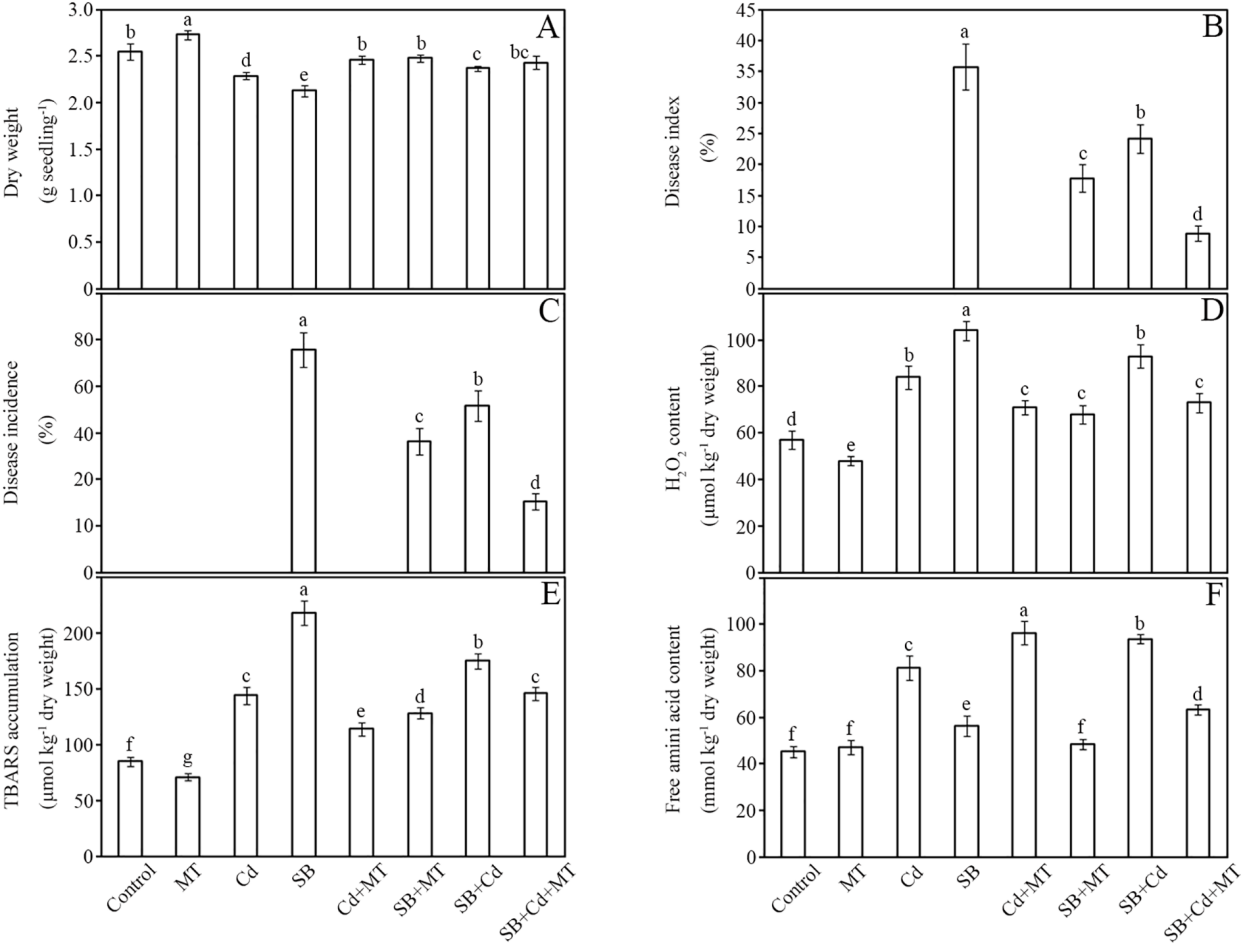
Dry weight, disease development and oxidative stress. Dry weight **(A)**, disease index **(B)**, disease incidence **(C)**, H_2_O_2_ content **(D)**, lipid peroxidation **(E)**, and free amino acid content **(F)** of peanut seedlings after treatment for 7 **(D)** Data are presented as mean ± SD (n = 5). Different letters indicate significant differences between treatments according to Duncan’s multiple range test (*p* < 0.05).

The effects of cadmium (Cd) and melatonin (MT) on peanut seedling growth, disease resistance (**Figure 1**), and oxidative stress responses were systematically evaluated (**Figure 2**). Compared to the control group (water treatment), application of melatonin alone (MT group) significantly promoted plant growth, increasing shoot dry weight by 13% (**Figure 2A**). In contrast, Cd stress (Cd group) and pathogen inoculation (SB group) significantly inhibited plant growth, reducing dry weight by 10% and 17%, respectively, after 7 days (**Figure 2A**). Notably, the application of melatonin under Cd stress (Cd+MT group) significantly reversed the growth inhibition caused by Cd alone, with the dry weight reduction ameliorated from 10% to just 2% (**Figure 2A**), underscoring melatonin’s direct protective role against Cd phytotoxicity. Furthermore, melatonin application (SB+MT group) mitigated pathogen-induced growth inhibition, showing only a 3% reduction in biomass. Combined treatments (SB+Cd and SB+Cd+MT) exhibited intermediate effects, with 7% and 5% reductions, respectively.

Disease resistance was markedly enhanced by melatonin. Compared to the pathogen-only group (SB), the SB+MT, SB+Cd, and SB+Cd+MT treatments reduced disease incidence index by 50%, 32%, and 75%, respectively (**Figure 2B**). Similarly, plant infection rates decreased by 41%, 25%, and 68% in these groups (**Figure 2C**).

Oxidative stress analysis revealed that Cd and pathogen stress significantly elevated H_2_O_2_ levels by 47% and 82%, respectively, compared to the control (**Figure 2D**). Melatonin application (SB+MT) substantially alleviated oxidative damage, limiting H_2_O_2_ accumulation to only 19% above control levels. Combined stress (SB+Cd) increased H_2_O_2_ by 63%, while SB+Cd+MT treatment reduced this to 28%. Lipid peroxidation followed similar trends, with Cd and pathogen stress increasing malondialdehyde (MDA) content by 69% and 156%, respectively, whereas melatonin-supplemented groups (SB+MT and SB+Cd+MT) showed 51% and 72% increases, indicating reduced oxidative damage (**Figure 2E**). Free amino acid content, a stress response marker, increased by 80% under Cd stress and 108% under combined SB+Cd stress, but melatonin treatments (SB+MT and SB+Cd+MT) moderated these increases to 25% and 40%, respectively (**Figure 2F**).

Cadmium accumulation patterns differed significantly between plant tissues (**Table 1**). Based on five biological replicates (n=5), roots accumulated 4.55-fold more Cd than shoots under Cd stress alone (523 ± 29 *vs*. 115 ± 7 mg kg^-1^ DW; *p* < 0.05). Pathogen co-exposure (+SB) altered Cd distribution, increasing root Cd by 21.8% (637 ± 22 *vs*. 523 ± 29 mg kg^-1^ DW; *p* < 0.05) while decreasing shoot accumulation by 36.9% (84 ± 5 *vs*. 115 ± 7 mg kg^-1^ DW; *p* < 0.05). Remarkably, melatonin supplementation (+SB+MT) reduced root and shoot Cd levels by 57% (272 ± 18 *vs*. 637 ± 22 mg kg^-1^ DW; *p* < 0.05) and 37% (53 ± 7 *vs*. 84 ± 5 mg kg^-1^ DW; *p* < 0.05), respectively, compared to Cd+SB treatment, demonstrating its role in Cd detoxification and translocation regulation.

**Table 1:** Cadmium (Cd) accumulation in peanut seedling.

	Control	Cd	Cd+SB	Cd+SB+MT
Root	0	523 ± 29b	637 ± 22a	272 ± 18c
shoot	0	115 ± 7d	84 ± 5e	53 ± 7f

Effects of disease and disease + melatonin combination treatment on Cd accumulation in root and shoot of peanut seedlings (mg kg^-1^ dry weight). Data are presented as mean ± SD (n = 5). Different letters indicate significant differences between treatments within each tissue according to Duncan’s multiple range test (*p* < 0.05). SB, southern blight; MT, melatonin.

### 3.3 Defense enzyme activities

**Figure 3:**
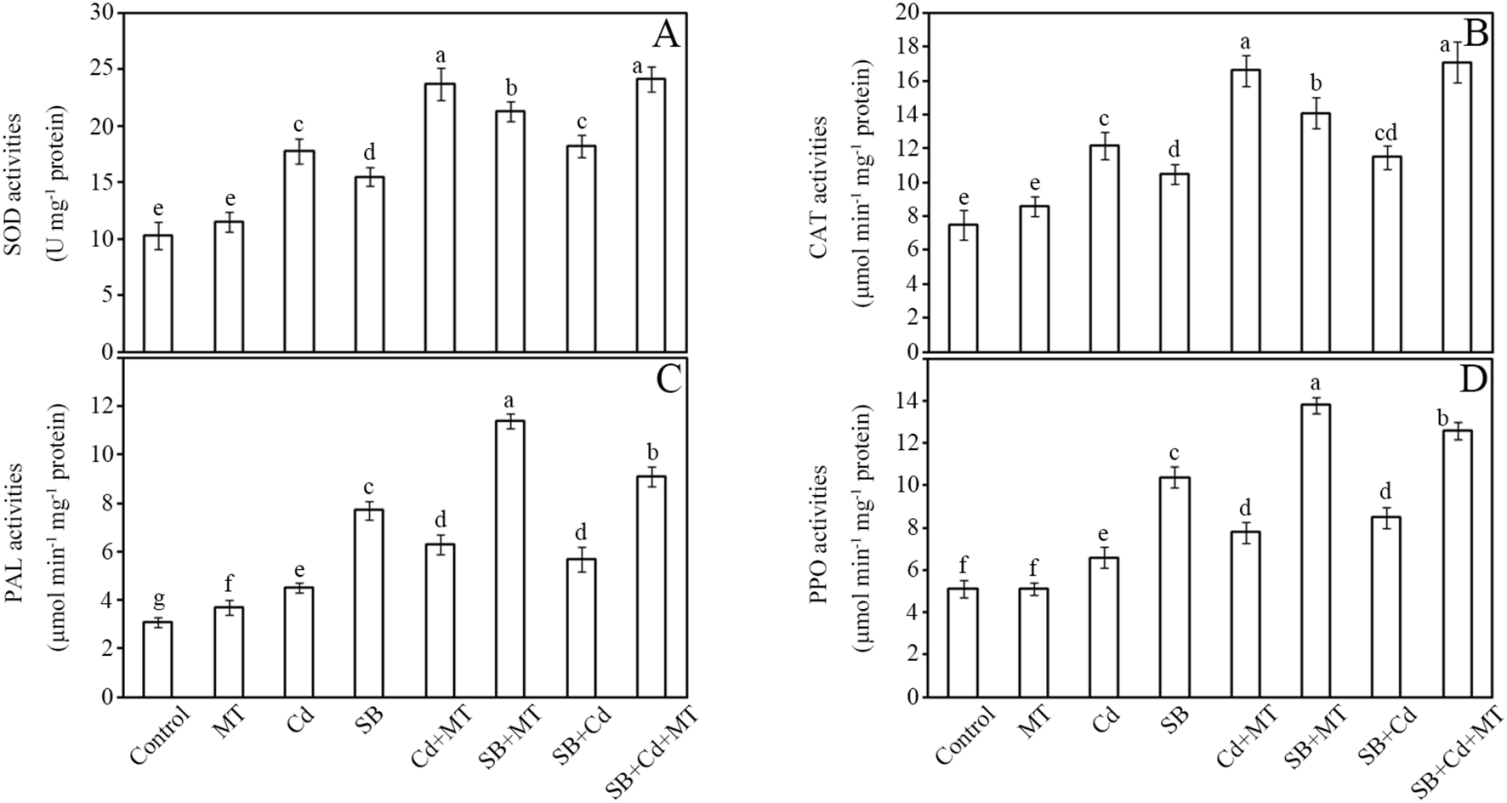
Defense enzyme activities. Activities of superoxide dismutase (SOD) **(A)**, catalase (CAT) **(B)**, phenylalanine ammonia lyase (PAL) **(C)** and polyphenol oxidase (PPO) **(D)** of stems in peanut seedlings after treatment for 7 **(D)** Data are presented as mean ± SD (n = 5). Different letters indicate significant differences between treatments according to Duncan’s multiple range test (*p* < 0.05).

The impacts of cadmium (Cd) and melatonin (MT) on key defense enzymes—SOD, CAT, PAL, and PPO—were systematically analyzed in peanut seedlings (**Figure 3**). Antioxidant enzyme activities (SOD and CAT) showed significant upregulation under stress conditions. Compared to the control group, Cd stress (Cd group) and pathogen inoculation (SB group) increased SOD activity by 73% and 50%, respectively, while melatonin application alone (MT group) induced a moderate increase of 12%. Notably, the combined treatment of Cd and melatonin (Cd+MT) resulted in a substantial enhancement of SOD activity (130% increase compared to control), while melatonin application under pathogen stress (SB+MT group) further enhanced this response to 107% (**Figure 3A**). Similarly, CAT activity rose by 63% (Cd group) and 40% (SB group), with melatonin alone (MT group) showing a 15% increase. The Cd+MT treatment significantly boosted CAT activity to 121% above control levels, while the SB+MT treatment increased activity to 88%. Combined stresses (SB+Cd) induced intermediate increases (SOD: 77%; CAT: 53%), whereas melatonin supplementation (SB+Cd+MT) maximized enzyme activation (SOD: 135%; CAT: 128%).

Disease-related enzymes (PAL and PPO) exhibited more pronounced responses. Pathogen challenge (SB group) triggered the strongest induction of PAL activity (148% increase), followed by Cd stress (45%) (**Figure 3C**). Melatonin application alone (MT group) increased PAL activity by 19%, while Cd+MT treatment enhanced it by 103%. Notably, melatonin treatment under pathogen stress (SB+MT group) amplified PAL activity to 268%, nearly doubling the pathogen-only response. PPO activity followed a similar pattern, with MT alone showing no significant change, while Cd+MT treatment increased PPO activity by 53% compared to control. The SB+MT group showed 171% enhancement versus 104% in the SB group (**Figure 3D**). Under combined stress (SB+Cd), PAL and PPO activities increased by 84% and 67%, respectively, while melatonin co-treatment (SB+Cd+MT) further elevated these levels to 194% (PAL) and 147% (PPO).

These results demonstrate that melatonin not only potentiates antioxidant defenses but also synergistically enhances pathogen-responsive enzymes, with the most robust effects observed under combined stress conditions. Furthermore, melatonin application significantly enhanced defense enzyme activities under Cd stress alone, indicating its active role in activating defense responses even in the absence of pathogen challenge.

### 3.4 Antioxidant profiles and redox status

**Figure 4:**
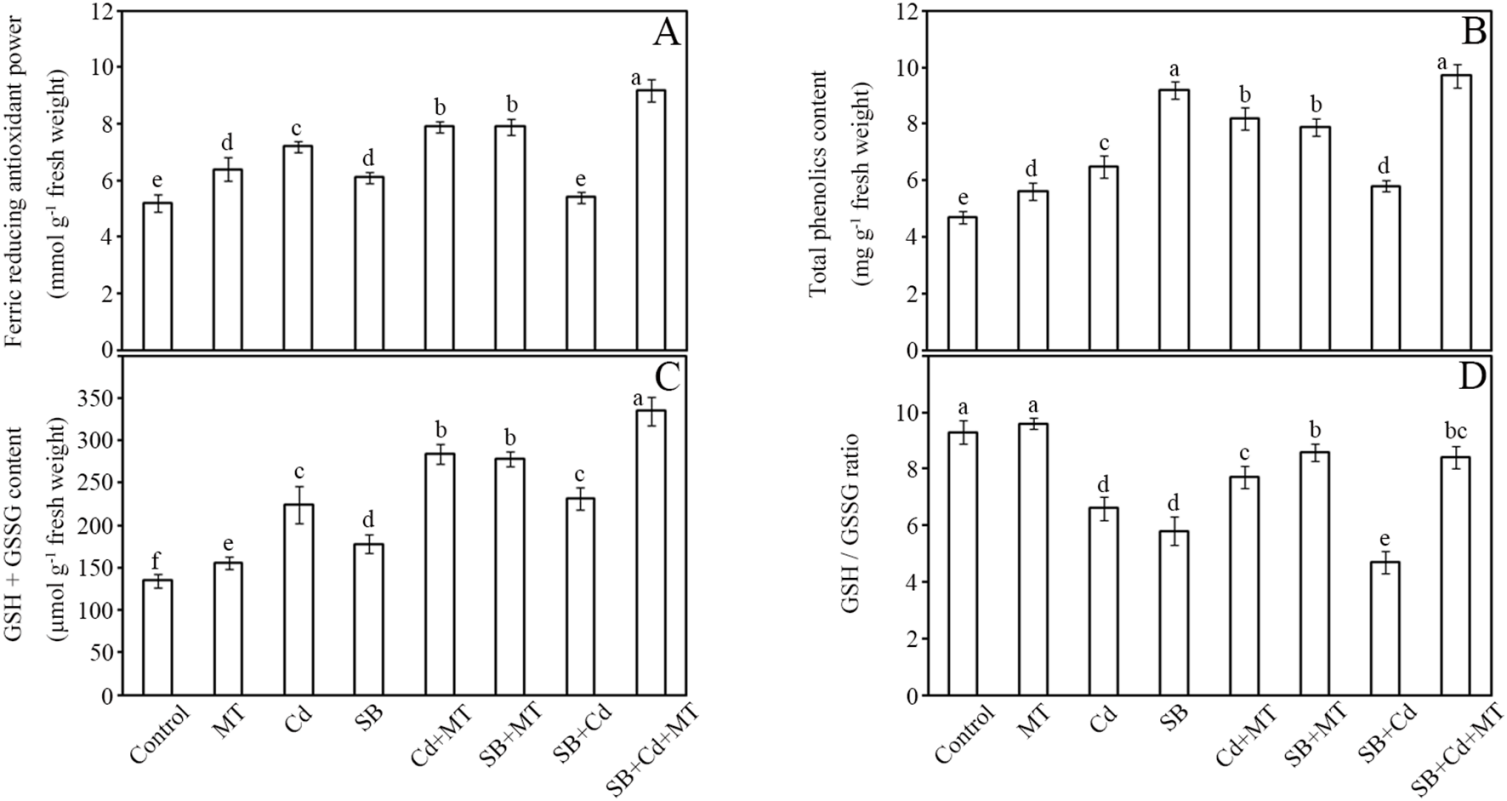
Antioxidants and redox ratio. Total antioxidant capacity **(A)**, total phenolics content **(B)**, total glutathione content **(C)**, and GSH/GSSG **(D)** of stems in peanut seedlings after treatment for 7 **(D)** Data are presented as mean ± SD (n = 5). Different letters indicate significant differences between treatments according to Duncan’s multiple range test (*p* < 0.05).

The effects of Cd and MT on antioxidant systems in peanut seedlings were comprehensively evaluated (**Figure 4**). Our results revealed distinct patterns in total antioxidant capacity (TAC), polyphenol content, glutathione pool, and redox homeostasis under different treatment conditions. Total antioxidant capacity showed progressive enhancement across treatments (**Figure 4A**).

Compared to the control, melatonin application alone (MT group) increased TAC by 23%. Cd stress (Cd group) increased TAC by 38%, while pathogen challenge (SB group) induced a 17% increase. Notably, the combined treatment of Cd and melatonin (Cd+MT) further enhanced TAC by 52% compared to control. Melatonin application under pathogen stress (SB+MT group) amplified this response to 52%. The combined stress (SB+Cd group) showed minimal change (4% increase), but melatonin co-treatment (SB+Cd+MT group) produced the strongest effect (77% increase).

Polyphenol accumulation exhibited treatment-specific dynamics (**Figure 4B**). Melatonin alone (MT group) increased polyphenol content by 19%. The SB group showed the highest induction (96% increase) among single-stress treatments, while Cd stress resulted in a 38% increase. The Cd+MT treatment significantly enhanced polyphenol accumulation to 74% above control levels. Interestingly, melatonin application (SB+MT) moderated the pathogen-induced response to 68%. Under combined stress (SB+Cd group), polyphenol content increased by 23%, whereas melatonin supplementation (SB+Cd+MT group) boosted this to 106%.

Glutathione metabolism displayed striking treatment effects (**Figure 4C**). Melatonin treatment alone (MT group) increased total glutathione content by 16%. Total glutathione (GSH+GSSG) content increased by 66% (Cd group) and 32% (SB group) relative to control. Notably, Cd+MT treatment dramatically enhanced glutathione accumulation to 110% above control levels. Melatonin treatment (SB+MT group) more than tripled this response (106% increase). The combined stress (SB+Cd group) elevated glutathione by 72%, with melatonin co-treatment (SB+Cd+MT group) achieving maximal accumulation (148% increase).

Redox homeostasis, as indicated by the GSH/GSSG ratio, showed significant perturbations (**Figure 4D**). Melatonin application alone (MT group) maintained a stable redox state with a slight increase (3%) in GSH/GSSG ratio. All other treatments reduced the ratio compared to control, with Cd stress alone decreasing it by 29%, while Cd+MT treatment showed a 17% reduction. The most severe declines occurred in the SB+Cd group (49% reduction) and SB group (38% reduction). Importantly, melatonin application substantially mitigated these effects, maintaining higher ratios in both SB+MT (8% reduction) and SB+Cd+MT (10% reduction) groups compared to their non-melatonin counterparts.

These findings demonstrate that melatonin differentially regulates various antioxidant components, with particularly strong effects on glutathione metabolism and redox balance maintenance under combined stress conditions. Moreover, melatonin application alone or in combination with Cd stress significantly enhanced the non-enzymatic antioxidant capacity, indicating its crucial role in reinforcing the cellular defense system.

### 3.5 JA and ABC transporter-related gene expression

To investigate the potential mechanism by which melatonin enhances the resistance of peanut seedlings to combined Cd and fungal (*Sclerotium rolfsii*) stress, we analyzed the expression patterns of four key genes in the stem tissues over a 7-day treatment period using qRT-PCR. As illustrated in **Supplementary Figure S1**, exogenous application of MT significantly up-regulated the transcript levels of two JA biosynthesis-related genes, *AhLOX7* and *AhOPR3*, as well as two Cd-transport-related genes, *ABCC3* and *ABCC4*, under dual stress conditions.

Specifically, in the control group (Cd + disease, CK), the expression of *AhLOX7* (**Supplementary Figure S1a**) increased initially, peaking at 3 days (3.5-fold), and then gradually decreased. In contrast, melatonin treatment resulted in a much higher induction, with expression reaching 6.2-fold at day 3 and remaining elevated at 4.3-fold by day 7. Similarly, *AhOPR3* expression (**Supplementary Figure S1b**) in the melatonin-treated group showed a substantial increase, with a maximum of 7.5-fold at day 3 and sustained high expression (5.0-fold at day 7), compared to the peak of 4.0-fold in the control.

For the heavy metal transporter genes, *ABCC3* (**Supplementary Figure S1c**) expression in the control peaked at 3.5-fold on day 5 and declined thereafter, while the MT group showed stronger up-regulation, reaching 5.2-fold on day 5 and maintaining 4.0-fold on day 7. Likewise, *ABCC4* (**Supplementary Figure S1d**) expression was enhanced under MT treatment, with a maximum value of 5.8-fold on day 5 compared to 3.8-fold in the control, and remained significantly higher at the end of the treatment period.

These results suggest that melatonin amplifies the JA signaling pathway and enhances the expression of ABCC-type transporters, which may contribute to reduced Cd accumulation and alleviated disease symptoms, thereby improving plant growth under combined stress.

### 3.6 Metabolomic profiling and multivariate analysis

LC-MS-based untargeted metabolomics revealed distinct metabolic alterations in peanut seedlings under different treatments. Using an ACQUITY UPLC BEH C18 column, we achieved comprehensive separation of the peanut metabolome within 16 min, detecting 11,036 metabolites in positive ionization mode and 6,275 metabolites in negative ionization mode (**Table 2**; **Supplementary Table S3**). The base peak chromatograms (**Supplementary Figure S2**) demonstrated significant metabolic divergence between samples designated as Jing_A (SB+Cd treatment) and Jing_B (SB+Cd+MT treatment).

**Table 2:** Metabolite analysis in positive and negative ion modes.

	Total metabolites	Up	Down	Total DE
Positive ion modes	11036	2075	228	2303
Negative ion modes	6275	839	398	1237

Multivariate statistical analysis further elucidated treatment-specific metabolic patterns. Unsupervised PCA showed clear separation between groups along principal components PC1 and PC2, explaining 23.1% (positive mode) and 28.1% (negative mode) of total variance (**Supplementary Figures S3a**, **S4a**). This confirmed distinct metabolic reprogramming induced by melatonin supplementation under combined stress (SB+Cd+MT).

Supervised PLS-DA and OPLS-DA models exhibited excellent predictive power, with R2Y/Q2 values of 0.999/0.834 (PLS-DA, positive mode) and 0.995/0.894 (PLS-DA, negative mode) for Jing_A/Jing_B comparisons (**Supplementary Figures S3b**, **S4b**). The OPLS-DA models showed similarly high reliability (R2Y/Q2 = 0.999/0.784 in positive mode; 0.995/0.77 in negative mode; **Supplementary Figures S3c**, **S4c**). Rigorous 7-fold cross-validation and permutation testing (n=100) confirmed model robustness, yielding intercepts of R2 = 0.99/Q2 = 0.1 (positive mode) and R2 = 0.98/Q2 = 0.16 (negative mode) (**Supplementary Figures S3d**, **S4d**).

These results demonstrate that LC-MS metabolomics coupled with multivariate analysis effectively discriminates metabolic signatures associated with Cd-pathogen co-stress and melatonin-mediated mitigation, providing a foundation for identifying key regulatory metabolites. All significant metabolites (*p* < 0.05; FC < 0.67 or > 1.5) were annotated using authentic standards with level 2+ confidence (MSI guidelines, **Supplementary Table S3**; **Supplementary Figure S5**).

### 3.7 Key differential metabolites in peanut stem

**Figure 5:**
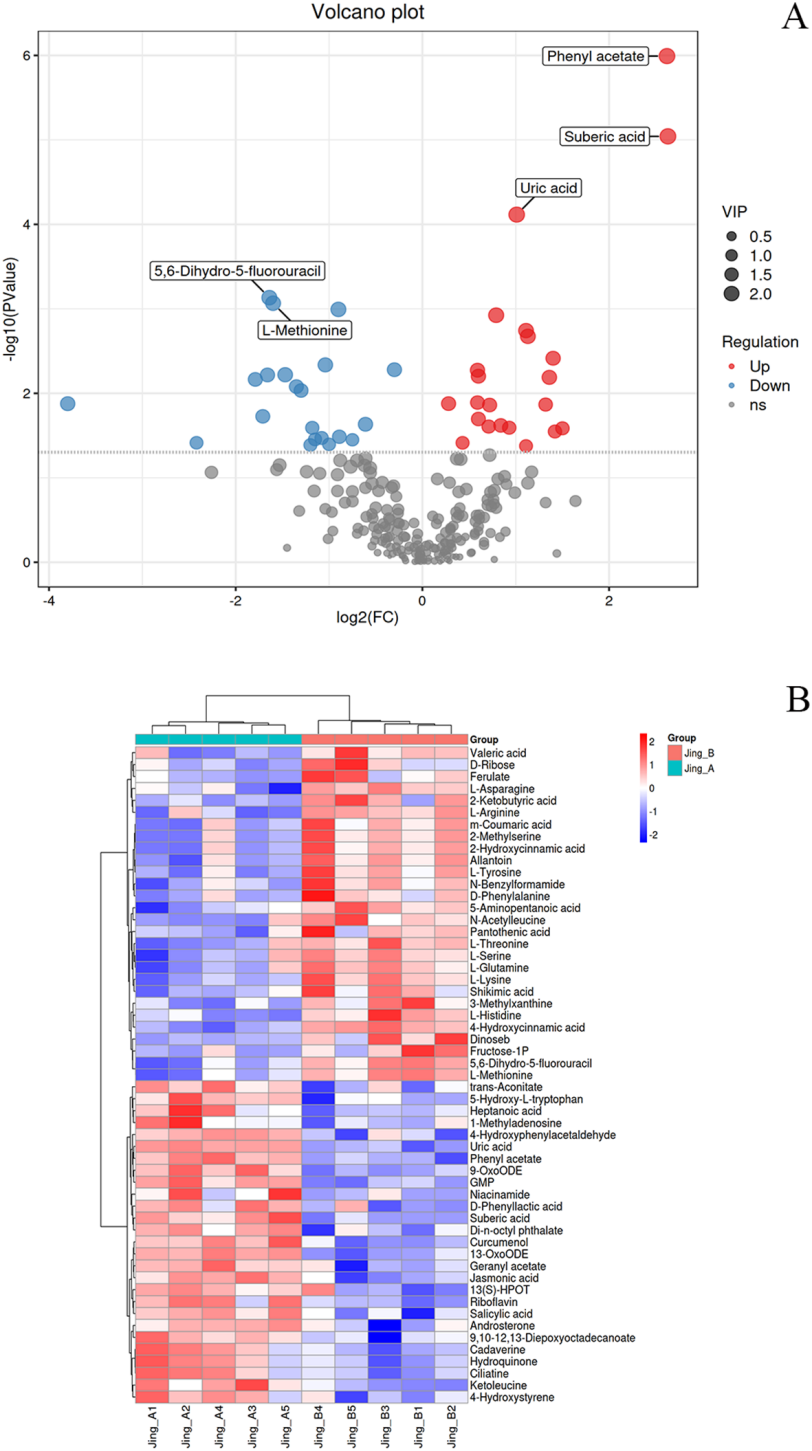
Differential Metabolite Analysis and Clustering Patterns in Peanut Seedlings under Combined Stress with Melatonin Treatment. **(A)** Volcano plot of differentially accumulated metabolites. The vertical dashed lines indicate the fold-change (FC) thresholds (|log_2_FC| > 1), and the horizontal dashed line represents the statistical significance threshold (-log_10_(*p*-value) > 1.3, equivalent to *p* < 0.05). Metabolites in the upper-right and upper-left quadrants (red and blue dots, respectively) are considered significantly upregulated or downregulated. Key defense-related metabolites are explicitly labeled, including suberic acid and uric acid. **(B)** Hierarchical clustering heatmap of significantly altered metabolites (based on thresholds in A). Each row represents a metabolite, and each column represents a biological replicate from different treatment groups. The color scale indicates normalized abundance levels (Z-score), with red denoting higher abundance and blue denoting lower abundance relative to the mean.

Non-targeted metabolomics analysis of peanut seedling stems subjected to dual stress revealed significant metabolic reprogramming induced by melatonin treatment (MT), as evidenced by numerous differentially expressed metabolites (DEMs) meeting stringent thresholds (|log_2_FC| ≥ 0.585 [FC >1.5 or <0.67], VIP >1, FDR <0.05) (**Supplementary Tables S4** ,**S5**). These differential metabolites were further visualized via volcano plot and hierarchical clustering heatmap (**Figure 5**). Regarding primary versus secondary metabolism, melatonin predominantly upregulated key secondary metabolites involved in stress defense. Notably, the stress-signaling phytohormone jasmonic acid (JA, FC = 1.73, VIP = 2.09, FDR = 0.0012) and the antimicrobial diterpenoid capsidiol (FC = 1.13, VIP = 1.69, FDR = 0.0129) were significantly elevated, indicating activation of defense pathways. Conversely, several primary metabolites, especially amino acids, were downregulated, including L-Proline (FC = 0.21, VIP = 1.29, FDR = 0.086), L-Methionine (FC = 0.33, VIP = 2.04, FDR = 0.00086), and L-Tyrosine (FC = 0.31, VIP = 1.69, FDR = 0.0187), suggesting potential resource reallocation towards defense synthesis or altered nitrogen metabolism under melatonin-mediated stress adaptation. Classifying DEMs biochemically revealed distinct trends: Sugar metabolism showed mixed responses, with significant downregulation of fructose-1-phosphate (FC = 0.19, VIP = 1.40, FDR = 0.0385) but upregulation of L-Erythrulose (FC = 2.49, VIP = 1.60, FDR = 0.0136). Lipids exhibited substantial modulation, with marked accumulation of the dicarboxylic acid suberic acid (FC = 6.18, VIP = 2.35, FDR = 9.1e-06), potentially linked to suberin deposition for barrier formation, and key oxylipins like 9-OxoODE (FC = 1.51, VIP = 1.82, FDR = 0.0053) involved in signaling. Amino acids were overwhelmingly downregulated (e.g., L-Histidine FC = 0.49, VIP = 1.90, FDR = 0.0046; L-Lysine FC = 0.41, VIP = 1.69, FDR = 0.0093; L-Aspartic acid FC = 0.54, VIP = 1.64, FDR = 0.0327), except for minor increases like L-Valine (FC = 1.85, VIP = 0.56, FDR = 0.43). Nucleic acid related metabolites included upregulated antioxidants like uric acid (FC = 2.01, VIP = 2.28, FDR = 7.7e-05) and downregulated salvage pathway components like cytidine (FC = 1.69, VIP = 0.87, FDR = 0.18).

Melatonin treatment elicited a comprehensive metabolic reprogramming in peanut seedlings under combined stress, with three key defense mechanisms being markedly enhanced (**Supplementary Table S5**). For disease resistance, the phytohormones jasmonic acid (FC = 1.73, VIP = 1.82, FDR = 0.05) and salicylic acid (FC = 1.64, VIP = 1.61, FDR = 0.14) were most strongly induced as core defense signals, supported by oxylipins 9-OxoODE (FC = 1.83, VIP = 1.92, FDR = 0.02), 13-OxoODE (FC = 1.70, VIP = 1.99, FDR = 0.01) and 13(S)-HPOT (FC = 1.56, VIP = 1.41, FDR = 0.21). The antioxidant system was significantly fortified by riboflavin (FC = 1.81, VIP = 1.77, FDR = 0.07), uric acid (FC = 2.29, VIP = 2.04, FDR = 0.02) and hydroquinone (FC = 2.11, VIP = 1.66, FDR = 10), with 5-hydroxy-L-tryptophan (FC = 1.56, VIP = 1.62, FDR = 0.11) contributing as a key precursor. Regarding Cd detoxification, trans-aconitate (FC = 1.66, VIP = 1.56, FDR = 0.17) and suberic acid (FC = 6.18, VIP = 1.88, FDR = 0.03) emerged as primary chelators, while ciliatine (FC = 2.09, VIP = 1.66, FDR = 0.10) provided additional metal-binding capacity. This coordinated metabolic response demonstrates melatonin’s ability to activate multi-layered defense pathways against combined biotic and abiotic stresses.

### 3.8 Integrated metabolic rewiring in peanut stem

**Figure 6:**
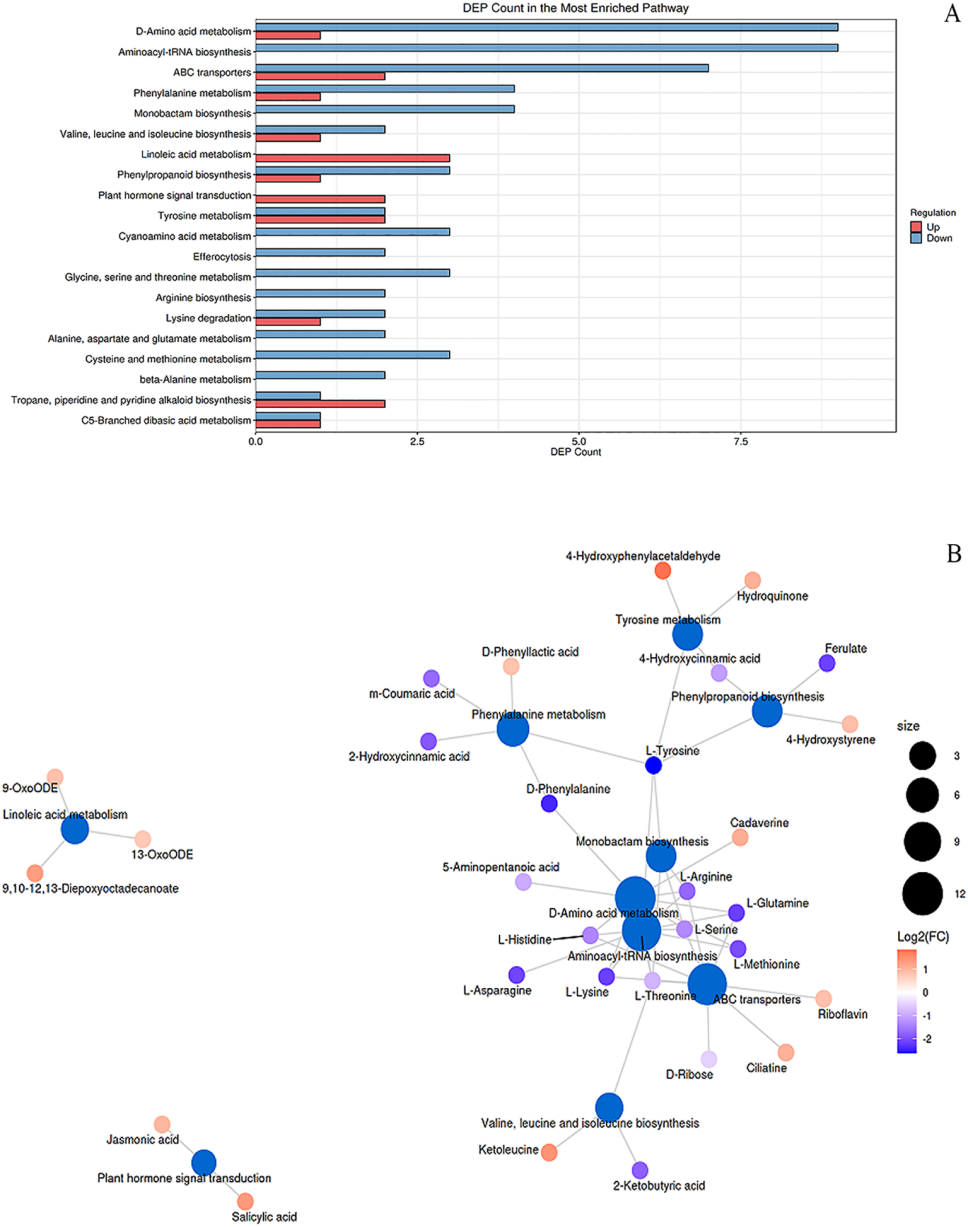
KEGG enrichment and metabolic network analysis of differential pathways in peanut seedlings. (**A**) Enrichment analysis of differential metabolic pathways in stems under Cd stress + MT treatment (SB + Cd + MT *vs*. SB + Cd). X-axis: number of differential metabolites; Y-axis: metabolic pathways. Red/blue: upregulated/downregulated metabolites. **(B)** Metabolic network: blue nodes = pathways (size indicates connected metabolite count); other nodes = metabolites. Red/blue: up-/downregulation. SB, Southern Blight; MT, melatonin.

Melatonin treatment induced significant metabolic reprogramming in peanut seedlings under combined stress (**Figure 6**; **Supplementary Table S6**), with distinct modulation of primary metabolism (e.g., Aminoacyl-tRNA biosynthesis, Hits=7, FDR = 1.26e-06, Impact=0.11; Arginine biosynthesis, Hits=3, FDR = 0.006, Impact=0.11) and secondary metabolism (e.g., Phenylpropanoid biosynthesis, Hits=2, FDR = 0.12, Impact=0.02; Isoquinoline alkaloid biosynthesis, Hits=3, FDR = 0.12, Impact=0.01). Carbohydrate metabolism was altered via Carbon fixation (Hits=1, FDR = 0.20, Impact=0.002), while lipid metabolism showed activation of Linoleic acid (Hits=3, FDR = 0.009, Impact=0.03) and α-Linolenic acid pathways (Hits=2, FDR = 0.11, Impact=0.16), the latter producing jasmonic acid (C08491, red). Nucleotide metabolism was perturbed in Purine metabolism (Hits=3, FDR = 0.11, Impact=0.06), with uric acid (C00366, red) upregulated. Energy metabolism was impacted via mTOR signaling (Hits=1, FDR = 0.11, Impact=0.25). For disease resistance, α-Linolenic acid metabolism (JA synthesis) and Phenylpropanoid biosynthesis (phenolics) were key. Antioxidant systems involved Riboflavin metabolism (Hits=1, FDR = 0.22, Impact=0.14) and Ascorbate metabolism (Hits=1, FDR = 0.35, Impact=0.003). Cd detoxification was linked to ABC transporters (Hits=7, FDR = 0.0004, Impact=0.05) and Ubiquinone biosynthesis (Hits=2, FDR = 0.14, Impact=0.001), suggesting enhanced metal chelation and efflux.

### 3.9 Melatonin enhances accumulation of key defense metabolites under dual stress

To elucidate the metabolic basis of melatonin-induced enhancement of resistance to combined Cd and *Sclerotium rolfsii* infection in peanut seedlings, we monitored the dynamic changes in the contents of three key metabolites—SA, JA, and suberic acid—in stem tissues over a 7-day period using ELISA. As shown in **Supplementary Figure S6**, exogenous application of 0.1 mM melatonin under dual stress conditions resulted in a remarkable accumulation of these defense-related metabolites compared to the stressed control (CK, Cd + disease).

In the control group, the SA content (**Supplementary Figure S6a**) increased over time, reaching 35-fold of the initial level (0 d) by day 7. Melatonin treatment dramatically enhanced this accumulation, with SA levels rising sharply to 50-fold at day 3 and reaching 85-fold by day 7, indicating a potentiation of SA-mediated defense signaling.

A similar amplifying effect was observed for JA (**Supplementary Figure S6b**). While the CK group showed a gradual increase to 30-fold at day 7, the melatonin group exhibited a much stronger induction, with JA content soaring to 43-fold at day 3 and maintaining a high level of 53-fold at the end of the treatment.

Notably, the content of suberic acid (**Supplementary Figure S6c**), which may play roles in both heavy metal detoxification and disease resistance, was also significantly boosted by melatonin. The control group reached a maximum of 28-fold on day 7, whereas the melatonin group showed an accelerated and sustained accumulation, peaking at 162-fold on day 7—an increase more than five-fold higher than the control.

These findings demonstrate that melatonin treatment strongly promotes the accumulation of salicylic acid, jasmonic acid, and suberic acid in peanut seedlings under combined stress, as accurately quantified by ELISA. The enhanced levels of these key metabolites correlate with the observed alleviation of disease symptoms and reduction in Cd toxicity, suggesting that melatonin primes multiple defense pathways to confer broad-spectrum resistance.

## 4 Discussion

### 4.1 Cd enhances peanut disease resistance alone

Despite 1 mM CdCl_2_ significantly inhibiting peanut seedling biomass (**Figure 2a**), its co-application with *Sclerotium rolfsii* (SB) reduced disease incidence by 32% compared to pathogen-only treatments (**Figures 1**, **2b, c**). This phenomenon aligns with reports that Cd may prime host defenses against necrotrophic pathogens rather than exert direct antifungal toxicity (Liu et al., 2022; Morkunas et al., 2018). Our physiological data support this priming hypothesis: Cd elevated free amino acids (**Figure 2f**), which serve as precursors for defense-related metabolites (He et al., 2019), and activated PAL/PPO enzymes (**Figures 3c–d**)—key regulators of phenolic biosynthesis (Taheri and Tarighi, 2010). This metabolic shift likely disrupts *S. rolfsii*’s dependency on host cell death mechanisms (Liu et al., 2025), as necrotrophs exploit dying tissues for nutrition (Cho, 2015). Furthermore, the elevated H_2_O_2_ levels induced by Cd (**Figure 2D**) may act as secondary messengers to fortify cell walls or induce defense gene expression, consistent with the role of reactive oxygen species in signaling plant immune responses against necrotrophic pathogens (Barna et al., 2012).

Notably, Cd-induced defense priming diverges from the “elemental defense” hypothesis (Dai et al., 2020), as peanuts showed minimal direct pathogen growth inhibition but significant biochemical adaptations. The sustained high ROS levels under Cd+SB (**Figure 2D**) suggest Cd’s role in defense signaling rather than antioxidative protection, evidenced by SOD/CAT activation (**Figures 3A–B**) and glutathione accumulation (**Figure 4C**). This aligns with heavy metals acting as stress signals to amplify immune responses (Morkunas et al., 2018). Furthermore, Cd-triggered lipid peroxidation (69% MDA increase; **Figure 2e**) may initiate the jasmonic acid signaling pathway, which plays a crucial role in defense against necrotrophic pathogens like *S. rolfsii* (Macioszek et al., 2023).

Additionally, Cd stress potentially enhances the biosynthesis of phenolic compounds and lignin through the phenylpropanoid pathway, creating physical barriers against pathogen invasion. This mechanism has been observed in other plant-pathogen systems where heavy metals induce structural defenses (Morkunas et al., 2018). The activation of these defense mechanisms represents a metabolic trade-off where plants redirect resources from growth to defense under combined stress conditions (Pandey et al., 2017).

In conclusion, Cd’s dual role as both stressor and defense primer depends on plant-pathogen interactions, with peanuts exhibiting adaptive resilience through biochemical rather than elemental mechanisms. This study highlights Cd’s capacity to “rewire” host metabolism against necrotrophic pathogens, though long-term trade-offs between growth and defense warrant further investigation.

### 4.2 Melatonin coordinately enhances cadmium detoxification and disease resistance

Melatonin-induced resistance to *Sclerotium rolfsii* under non-stressed conditions. Our results demonstrate that melatonin (MT) treatment significantly enhances peanut resistance to *Sclerotium rolfsii*, reducing disease severity by 50% (**Figures 2B, C**). This effect surpasses the 30–40% reductions reported for MT-induced resistance against *Botrytis cinerea* in *Arabidopsis* (Zhu et al., 2021) and *Ralstonia solanacearum* in tomato (Liu et al., 2024), suggesting peanut’s heightened sensitivity to MT-mediated defense priming. Notably, while earlier studies in Arabidopsis indicated that melatonin and salicylic acid (SA) may function synergistically in some biotic stress contexts (Hernández-Ruiz and Arnao, 2018), our work reveals a more pronounced and simultaneous upregulation of both JA and SA pathways specifically under combined Cd and fungal stress, highlighting a stress context-dependent mechanism. This is further supported by the significant upregulation of key metabolites including salicylic acid (FC = 1.64; ELISA: +57%), jasmonic acid (FC = 1.73; ELISA: +78%), and suberic acid (FC = 6.18; ELISA: +471%) in melatonin-treated plants under dual stress (**Supplementary Figure S6**), which are known to be involved in disease resistance and Cd chelation. For industrial peanut production, this MT-induced disease resistance is particularly valuable as it reduces reliance on chemical fungicides while maintaining crop yield and quality in pathogen-prone fields. Physiologically, MT triggered a robust antioxidant response, decreasing H_2_O_2_ accumulation by 77% (**Figure 2D**) and lipid peroxidation (MDA content) by 51% (**Figure 2E**), while elevating SOD and CAT activities by 107% and 88%, respectively (**Figures 3A, B**) – exceeding improvements observed in pepper (Kaya and Doganlar, 2019). The exceptional induction of phenylpropanoid pathway enzymes PAL (268%) and PPO (171%) (**Figures 3C, D**) underscores MT’s capacity to fortify phenolic biosynthesis for pathogen defense, consistent with prior findings in peanut (Khatediya et al., 2018).

Under Cd stress, MT’s defense strategy expanded to include hormonal and metabolic adjustments. Notably, MT treatment reduced Cd accumulation in peanut seedlings by 57% in roots and 37% in shoots (**Table 1**), despite Cd’s known antimicrobial properties (Liu et al., 2022; Dai et al., 2020). This Cd reduction capability is crucial for industrial applications, as it allows peanut cultivation in moderately Cd-contaminated soils while meeting food safety standards for heavy metal content. While Cd itself may contribute to disease suppression (**Figure 2**), MT likely compensates for reduced Cd uptake by (1) alleviating Cd-induced growth inhibition, thereby enabling the synthesis of more defense compounds, and (2) activating JA signaling (FC = 1.73; **Supplementary Table S3**) and other pathways to maintain disease resistance. This is consistent with studies showing that JA signaling reduces Cd uptake and translocation, as demonstrated in Arabidopsis (Lei et al., 2020) and rice (Zhang et al., 2025). In particular, melatonin significantly activated the expression of key genes involved in the JA biosynthesis pathway (*AhLOX7* and *AhOPR3*) and ABC transporter genes (*ABCC3* and *ABCC4*) (**Supplementary Figure S1**), which are critical for enhancing disease resistance and Cd detoxification (Yu et al., 2018; Ma et al., 2024). Metabolomic analyses revealed that MT simultaneously activated both jasmonic acid (JA, FC = 1.73) and salicylic acid (SA, FC = 1.64) pathways (**Supplementary Table S5**), contrasting with non-stressed plants where JA alone suffices for necrotrophic defense (Li et al., 2022). This coordinated induction of JA and SA under combined stress diverges from the model proposed by Shi et al. (2015) in Arabidopsis, where melatonin primarily enhanced the expression of CBF/DREB1 transcription factors to confer cross-protection against abiotic and biotic stresses. Our results suggest that in peanut, melatonin employs a more hormone-centric strategy, directly modulating JA and SA to achieve dual stress resilience, which may represent a species-specific or stress-combination adaptive response (Macioszek et al., 2023). This dual activation likely reflects an adaptation to co-occurring stresses: JA-mediated defense against *S. rolfsii* (Tiwari et al., 2021) and SA-mediated mitigation of Cd toxicity via antioxidant regulation, as demonstrated by Guo et al. (2019) who showed that salicylic acid signals plant defense against cadmium toxicity. Similar synergism was reported in tomato, where MT and fungal metabolites co-upregulated JA/SA pathways to enhance Cd tolerance and disease resistance (Liu et al., 2024). Metabolomic shifts further supported this integrated response. MT elevated riboflavin (FC = 1.81) and phthalic acid (FC = 2.16) (**Supplementary Table S3**), metabolites with dual roles in ROS scavenging and pathogen inhibition. Riboflavin’s association with JA-mediated defense was noted in rice (Taheri and Tarighi, 2010), while phthalic acid’s antifungal activity against *Fusarium* (Wu et al., 2015) suggests a novel mechanism for *S. rolfsii* suppression. MT also maintained redox homeostasis (GSH/GSSG ratio at 92% of control; **Figure 4D**), highlighting its role in balancing defense and detoxification. MT’s metabolic reprogramming under Cd+disease stress involved: 1) Energy reallocation: Suppression of amino acid biosynthesis (Aminoacyl-tRNA pathway, Hits=7, FDR = 1.26e-06; L-histidine FC = 0.49, L-methionine FC = 0.33; **Supplementary Tables S5**, **S6**), reducing membrane leakage (free amino acids decreased by 40%; **Figure 2F**) as observed in Cd-stressed plants (Zhu et al., 2018). 2) Membrane stabilization: Activation of linoleic acid metabolism (oleic acid pathway, Hits=3, FDR = 0.009; 9-OxoODE FC = 1.83, 13-OxoODE FC = 1.70; **Supplementary Tables S5**, **S6**), preserving cellular integrity (Mariutto et al., 2014). 3) Metal chelation: Upregulation of uric acid (Purine metabolism, FC = 2.01), which acts as an endogenous antioxidant to promote stress tolerance (Sun et al., 2021), and suberic acid (ABC transporters, FC = 6.18; Prévéral et al., 2009), the latter’s 6-fold increase correlating with reduced Cd translocation (r=−0.89, p<0.01). Specifically, the dramatic upregulation of suberic acid is of particular interest. As a dicarboxylic acid, suberic acid contains carboxyl groups that may chelate Cd^2+^ ions, potentially forming stable complexes and reducing Cd bioavailability (Sun et al., 2020; Luo et al., 2015). Similarly, uric acid has been shown to enhance Cd tolerance through antioxidant mechanisms and possibly metal complexation (Nourimand and Todd, 2016). Previous metabolomic studies on Cd hyperaccumulators, such as Sedum species, have reported significant accumulation of suberic acid under Cd exposure, supporting its potential role in metal detoxification (Sun et al., 2020; Luo et al., 2015). In our study, the strong negative correlation between suberic acid levels and Cd translocation suggests that melatonin-induced suberic acid may contribute to reduced Cd mobility, possibly through sequestration in root apoplast or vacuoles. However, we note that this proposed chelation mechanism remains speculative and is based on correlative data and molecular structure inference rather than direct experimental evidence such as *in vitro* binding assays. Further studies are needed to validate the binding affinity, stoichiometry, and in planta localization of suberic acid–Cd complexes. 4) Phenylpropanoid biosynthesis (Hits=2, FDR = 0.12) contributed to a 106% increase in polyphenols (**Figure 4B**), enhancing disease resistance (Larrauri et al., 2016).

MT’s dual stress mitigation bypasses the typical trade-off between abiotic and biotic responses (Ben Rejeb et al., 2014). While Cd alone increased H_2_O_2_ by 47% (**Figure 2D**), MT under combined stress reduced oxidative damage (MDA content 72% *vs*. 156% in pathogen-only; **Figure 2E**) and amplified SOD (135%) and CAT (128%) activities beyond additive effects (**Figures 3A, B**), likely via riboflavin and ascorbate metabolism (Hits=1, FDR = 0.22/0.35). The 148% increase in total glutathione (**Figure 4C**) and maintained GSH/GSSG ratio (**Figure 4D**) outperformed MT’s effects in drought-stressed pepper (Kaya and Doganlar, 2019), highlighting its utility in Cd-contaminated soils (yield losses >30%; Zhao et al., 2021). Hormonally, MT’s rebalancing (JA FC = 1.73, SA FC = 2.57) primed defenses without growth penalties seen in tomato (Liu et al., 2024), making it suitable for integration with low-Cd cultivars (Shi et al., 2014). The preferential induction of α-linolenic acid metabolism (Hits=2, FDR = 0.11) and phenylpropanoid pathways may also enhance the nutritional value of peanut byproducts (Larrauri et al., 2016).

### 4.3 Research limitation and future perspectives

While this study demonstrates melatonin’s dual role in Cd detoxification and disease resistance in peanut seedlings, several limitations warrant attention. First, untargeted metabolomics revealed correlations between melatonin and defense metabolites (e.g., jasmonic acid, suberic acid), but mechanistic validation through targeted metabolomics and transcriptomics is needed to elucidate regulatory networks in Cd sequestration (e.g., ABC transporters; Prévéral et al., 2009) and disease resistance (e.g., phenylpropanoid biosynthesis). Gene-edited peanut lines (e.g., PAL- or JA-deficient mutants), following approaches in other oilseeds (Shah et al., 2025), could provide causal evidence. Second, focusing on stems (primary infection site for *Sclerotium rolfsii*) neglects root-specific Cd responses; future studies should examine root adaptations (Bovet et al., 2005) and foliar detoxification for whole-plant resistance. Third, our study was conducted under controlled greenhouse conditions, which may not fully replicate field complexities such as soil microbial interactions, fluctuating Cd bioavailability, and variable climate. Future field trials in Cd-contaminated regions (e.g., China’s Yangtze River Delta; Wang et al., 2015) are essential to validate the efficacy of melatonin under real-world agricultural scenarios. Recent studies have demonstrated the positive effects of melatonin on crop productivity and stress resilience in field settings, such as in peanut (Li et al., 2024) and rice (Yang et al., 2025), supporting its practical potential. Fourth, while melatonin alone shows promise, its synergistic effects with other bioactive compounds (e.g., chitosan, Al-Saadi et al., 2025; zinc, Yavar et al., 2024) or biocontrol agents (e.g., *Trichoderma* spp.; Safari Motlagh et al., 2022) should be explored to develop integrated stress management strategies. Additionally, combining melatonin with low-Cd cultivars (Shi et al., 2014) may improve field efficacy, though metabolic trade-offs (e.g., amino acid downregulation; Hariharan et al., 2021) must be optimized for food/feed quality. Repurposing peanut byproducts (e.g., skins rich in phenolics; Larrauri et al., 2016) as biostimulants could enhance circular agriculture. Given peanut’s economic importance (Akram et al., 2018), future research should prioritize: (1) mechanistic validation via multi-omics, (2) field validation under real-world stresses, and (3) integration with circular agriculture for sustainable industrial application.

## 5 Conclusion

This study establishes melatonin as a dual-function protectant in peanut, simultaneously mitigating Cd toxicity and enhancing resistance to *Sclerotium rolfsii* through coordinated physiological and metabolic reprogramming. Our results indicate that melatonin orchestrates a multi-layered defense strategy by: (1) activating both JA and SA signaling pathways, as evidenced by the upregulation of key genes (*AhLOX7*, *AhOPR3*) involved in JA biosynthesis; (2) enhancing antioxidant systems (SOD/CAT/glutathione) to maintain redox homeostasis under combined stress; and (3) reducing Cd accumulation in shoots by 37%, potentially through the upregulation of ABC transporters (*ABCC3*, *ABCC4*) implicated in Cd sequestration and vacuolar compartmentalization. These findings enhance our understanding of plant stress cross-talk by revealing how melatonin integrates hormonal signaling with antioxidant and detoxification mechanisms to confer dual stress resilience. From an application perspective, melatonin treatment reduced disease incidence by 75% and shoot Cd content significantly, showing promise for improving both health and yield of peanut in contaminated soils. The 106% increase in polyphenols and 148% increase in glutathione further indicate a reinforced crop defense system under stress. This work provides a proof-of-concept for melatonin as a potential eco-friendly alternative to chemical fungicides and a sustainable strategy for mitigating heavy metal contamination. However, further studies are needed to validate these mechanisms under field conditions and to integrate multi-omics approaches for a systemic understanding of melatonin-mediated dual stress resistance.

## Data Availability

The original contributions presented in the study are included in the article/**Supplementary Material**. Further inquiries can be directed to the corresponding author.

## Supplementary material

Supplementary Table 1 Dry weight, disease index, and cadmium content in peanut seedlings subjected to combined cadmium and southern blight infection stress under different melatonin concentrations (0, 0.01, 0.1, and 1.0 mM) after 7 days of treatment. Data are presented as mean ± SD (n = 5). Different letters indicate significant differences among treatments according to Duncan’s multiple range test (p < 0.05).

Supplementary Table 2 Primer sequences used for qRT-PCR analysis of JA biosynthesis and Cd transporter genes in this study.

Supplementary Table 3 Total Metabolic Feature Peaks. Complete set of 17311 raw LC-MS features detected in untargeted metabolomics across both positive and negative ionization modes prior to annotation

Supplementary Table 4 Initially Annotated Metabolites. Initial annotation results showing 1509 identified metabolites (1062 positive mode 447 negative mode) before statistical filtering including both differential and non-differential compounds.

Supplementary Table 5 High Confidence Differential Metabolites. Final set of 232 statistically significant metabolites (147 positive mode 85 negative mode) meeting VIP ≥ 1 and *p* ≤ 0.05 criteria identified in both ionization modes.

Supplementary Table 6 Enriched KEGG Metabolic Pathways. Listing of 60 significantly enriched KEGG pathways with metabolic pathways.

Supplementary Figure 1 Time-course expression profiles of four key genes (*AhLOX7, AhOPR3, ABCC3, ABCC4*) in peanut stems under combined Cd and *S. rolfsii* stress with or without melatonin (MT) treatment. Gene expression was measured at 0, 1, 3, 5, and 7 days post-inoculation. Values are mean ± SD (n = 5). Different letters indicate significant differences (p < 0.05) between treatments at each time point.

Supplementary Figure 2 Base peak chromatogram of Jing_A and Jing_B samples in positive **(A)** and negative **(B)** ESI mode

Supplementary Figure 3 Multivariate statistical score scatter plots and permutation test between Jing_A and Jing_B in positive ion mode. **(A)** PCA; **(B)** PLS-DA; **(C)** OPLS-DA; **(D)** permutation test of the OPLS-DA model.

Supplementary Figure 4 Multivariate statistical score scatter plots and permutation test between Jing_A and Jing_B in negative ion mode. **(A)** PCA; **(B)** PLS-DA; **(C)** OPLS-DA; **(D)** permutation test of the OPLS-DA model.

Supplementary Figure 5 Identification of key differential metabolites using authentic standards. Chromatographic match confirms annotation of **(A)** L-Argine; **(B)** Shikimic acid; **(C)** Salicylic acid; **(D)** Ferulate; **(E)** 9-OxoODE; **(F)** Curcumene.

Supplementary Figure 6 Contents of salicylic acid, jasmonic acid, and suberic acid in stems. Salicylic acid **(A)**, jasmonic acid **(B)**, and suberic acid **(C)** contents in the stems of peanut seedlings under combined cadmium and southern blight stress after treatment with 0.1 mM melatonin for 0, 1, 3, 5 and 7 d. Data are presented as mean ± SD (n = 5). Different letters indicate significant differences among treatments according to Duncan’s multiple range test (p < 0.05).
