# Supplementary figures and images for: Dual defense: melatonin simultaneously mitigates cadmium toxicity and southern blight in peanut
Source: Front Plant Sci. 2025 Oct 16;16:1686151. doi: 10.3389/fpls.2025.1686151 (PMC12571740; doi:10.3389/fpls.2025.1686151)

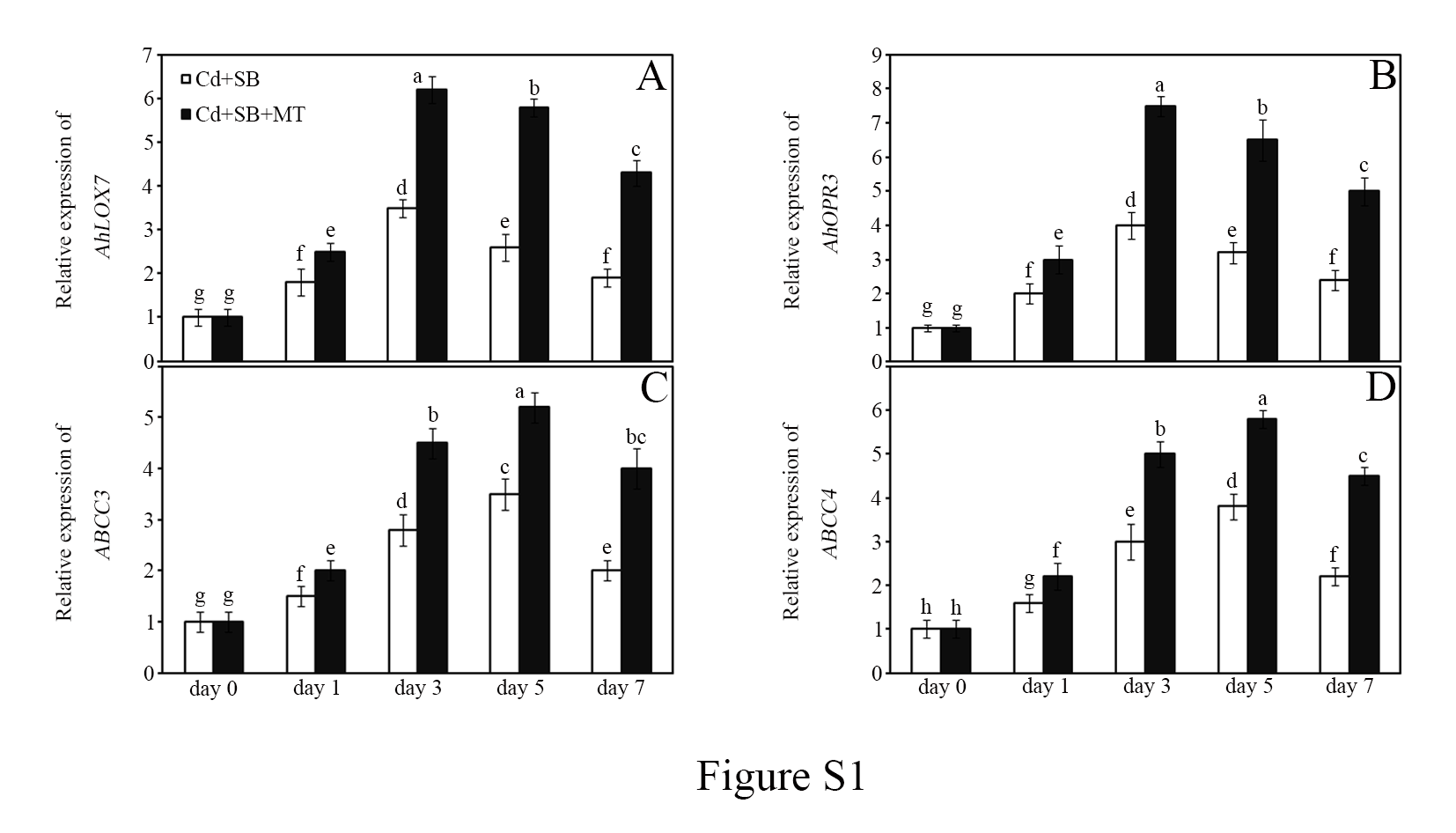

Supplement: Supplementary Table 1 — Dry weight, disease index, and cadmium content in peanut seedlings subjected to combined cadmium and southern blight infection stress under different melatonin concentrations (0, 0.01, 0.1, and 1.0 mM) after 7 days of treatment. Data are presented as mean ± SD (n = 5). Different letters indicate significant differences among treatments according to Duncan’s multiple range test (p < 0.05). [file DataSheet1.zip › Suppl Mater/Figure S1.tif]

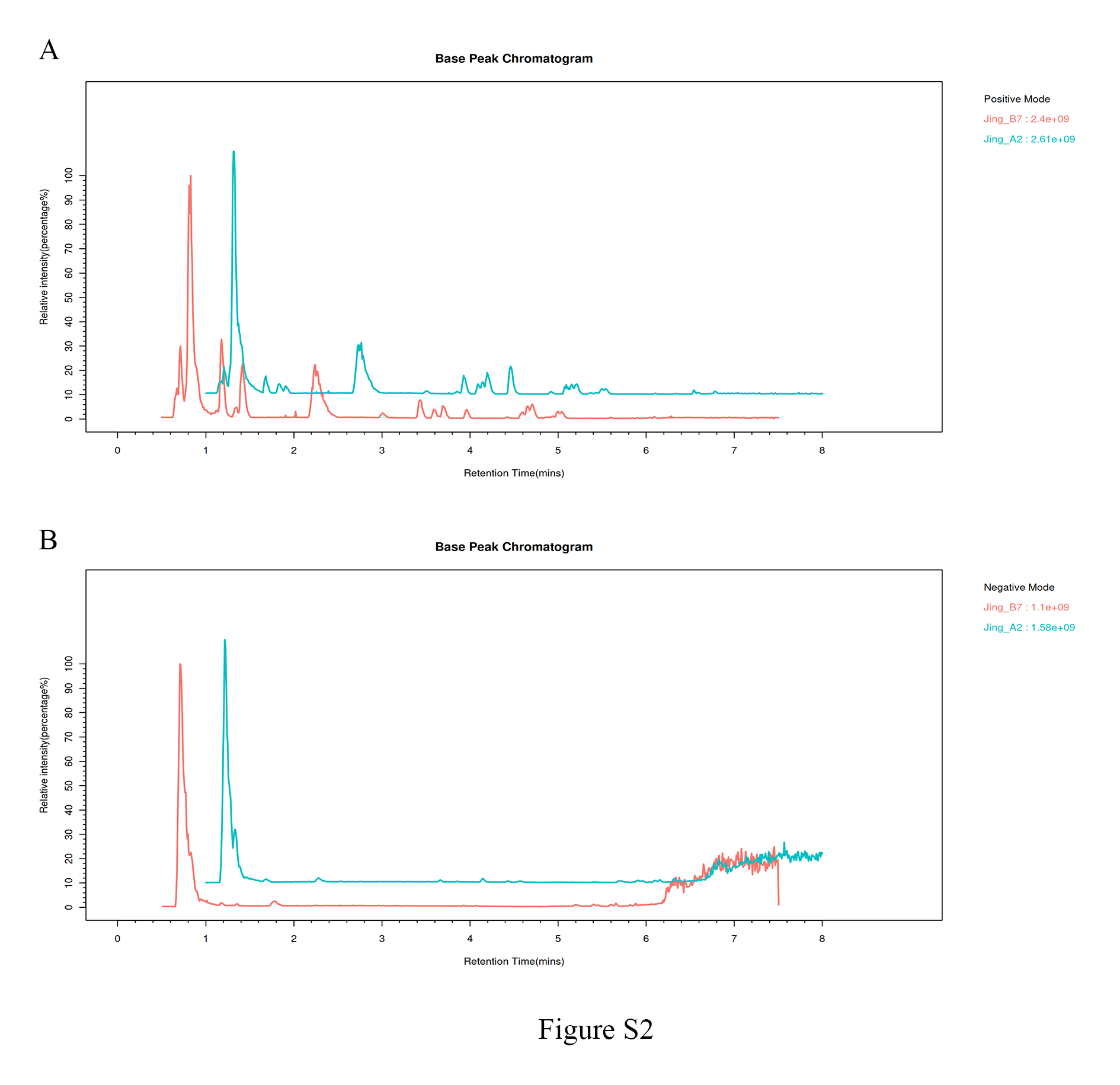

Supplement: Supplementary Table 1 — Dry weight, disease index, and cadmium content in peanut seedlings subjected to combined cadmium and southern blight infection stress under different melatonin concentrations (0, 0.01, 0.1, and 1.0 mM) after 7 days of treatment. Data are presented as mean ± SD (n = 5). Different letters indicate significant differences among treatments according to Duncan’s multiple range test (p < 0.05). [file DataSheet1.zip › Suppl Mater/Figure S2.tif]

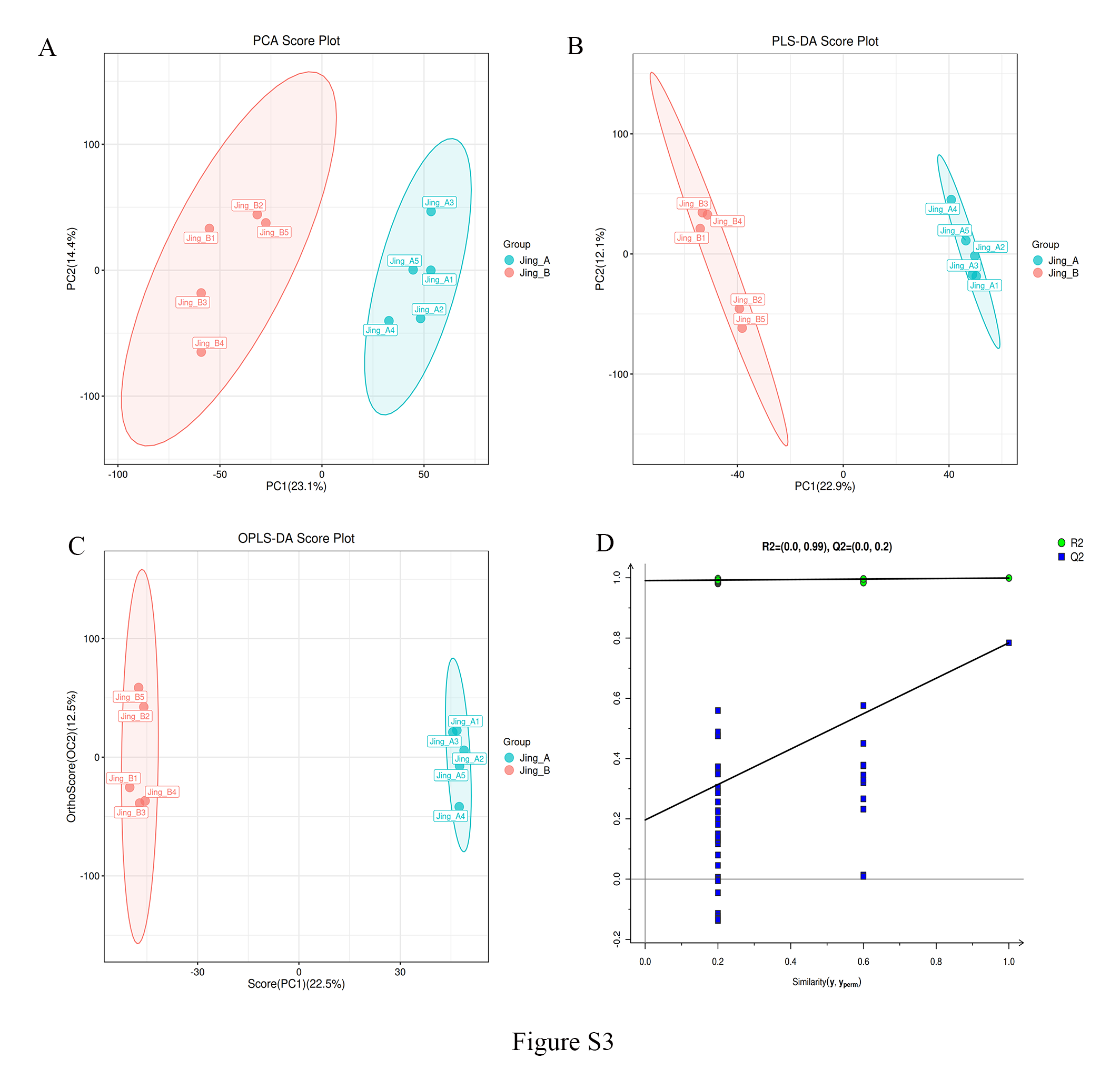

Supplement: Supplementary Table 1 — Dry weight, disease index, and cadmium content in peanut seedlings subjected to combined cadmium and southern blight infection stress under different melatonin concentrations (0, 0.01, 0.1, and 1.0 mM) after 7 days of treatment. Data are presented as mean ± SD (n = 5). Different letters indicate significant differences among treatments according to Duncan’s multiple range test (p < 0.05). [file DataSheet1.zip › Suppl Mater/Figure S3.tif]

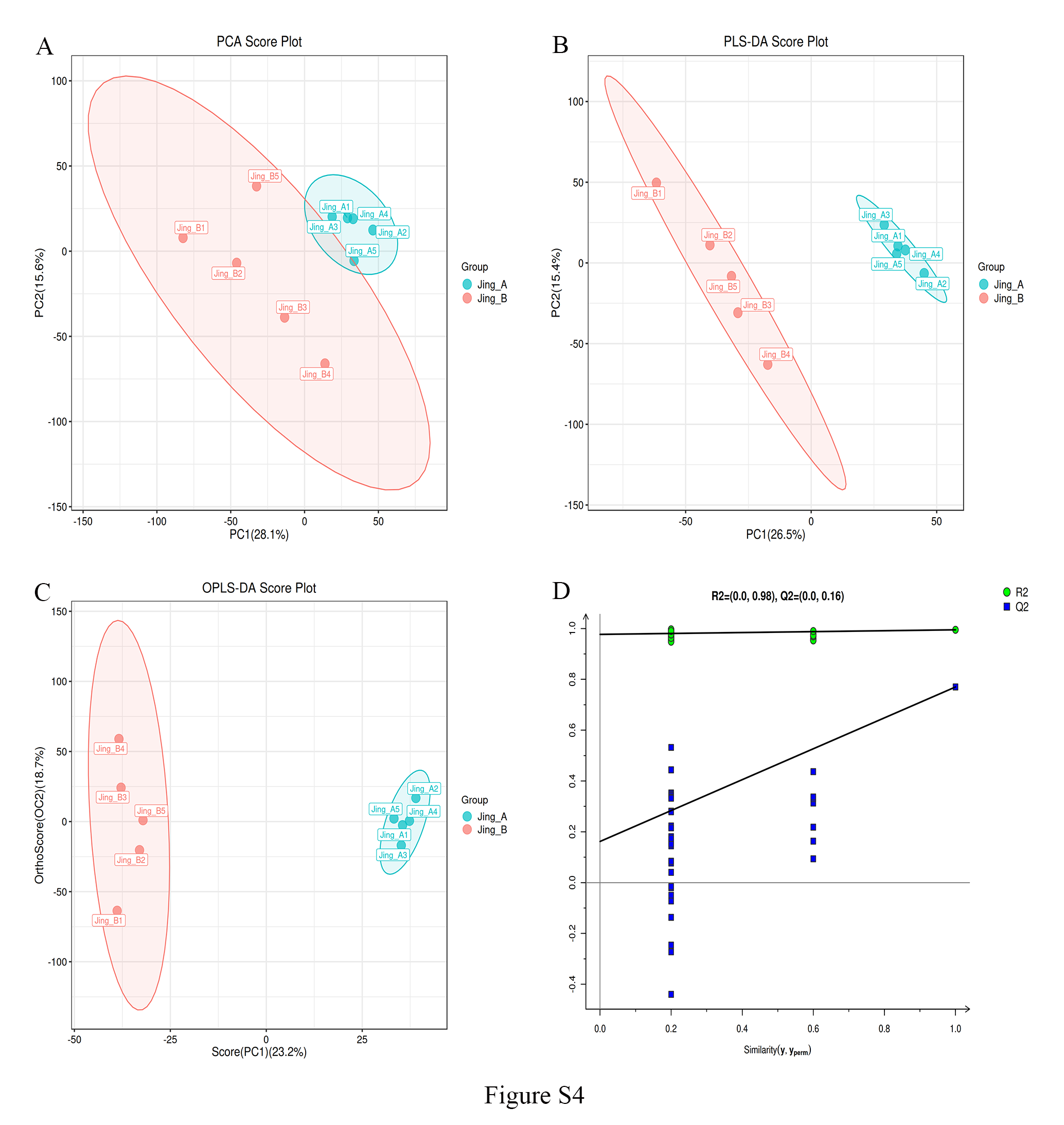

Supplement: Supplementary Table 1 — Dry weight, disease index, and cadmium content in peanut seedlings subjected to combined cadmium and southern blight infection stress under different melatonin concentrations (0, 0.01, 0.1, and 1.0 mM) after 7 days of treatment. Data are presented as mean ± SD (n = 5). Different letters indicate significant differences among treatments according to Duncan’s multiple range test (p < 0.05). [file DataSheet1.zip › Suppl Mater/Figure S4.tif]

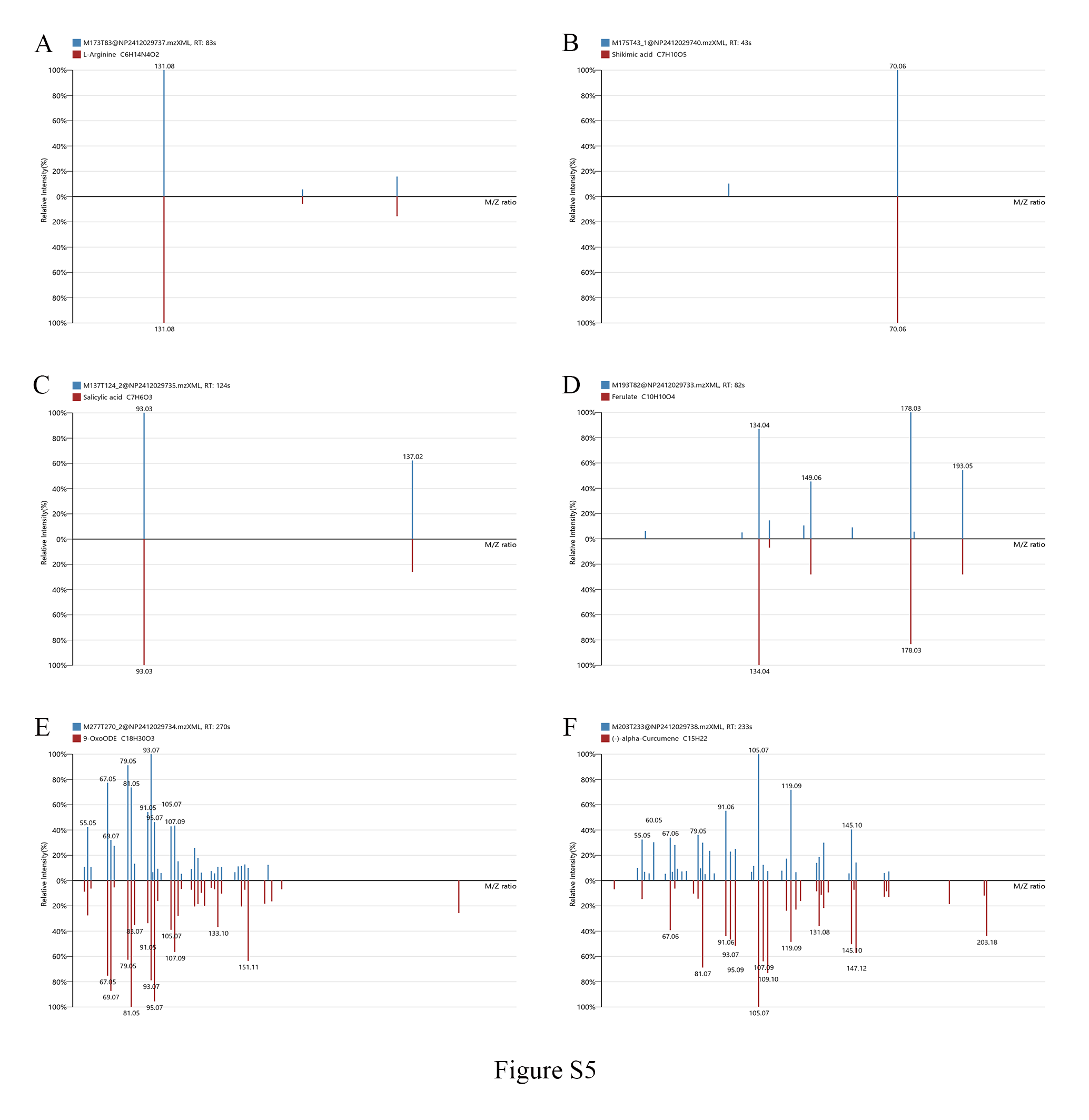

Supplement: Supplementary Table 1 — Dry weight, disease index, and cadmium content in peanut seedlings subjected to combined cadmium and southern blight infection stress under different melatonin concentrations (0, 0.01, 0.1, and 1.0 mM) after 7 days of treatment. Data are presented as mean ± SD (n = 5). Different letters indicate significant differences among treatments according to Duncan’s multiple range test (p < 0.05). [file DataSheet1.zip › Suppl Mater/Figure S5.tif]

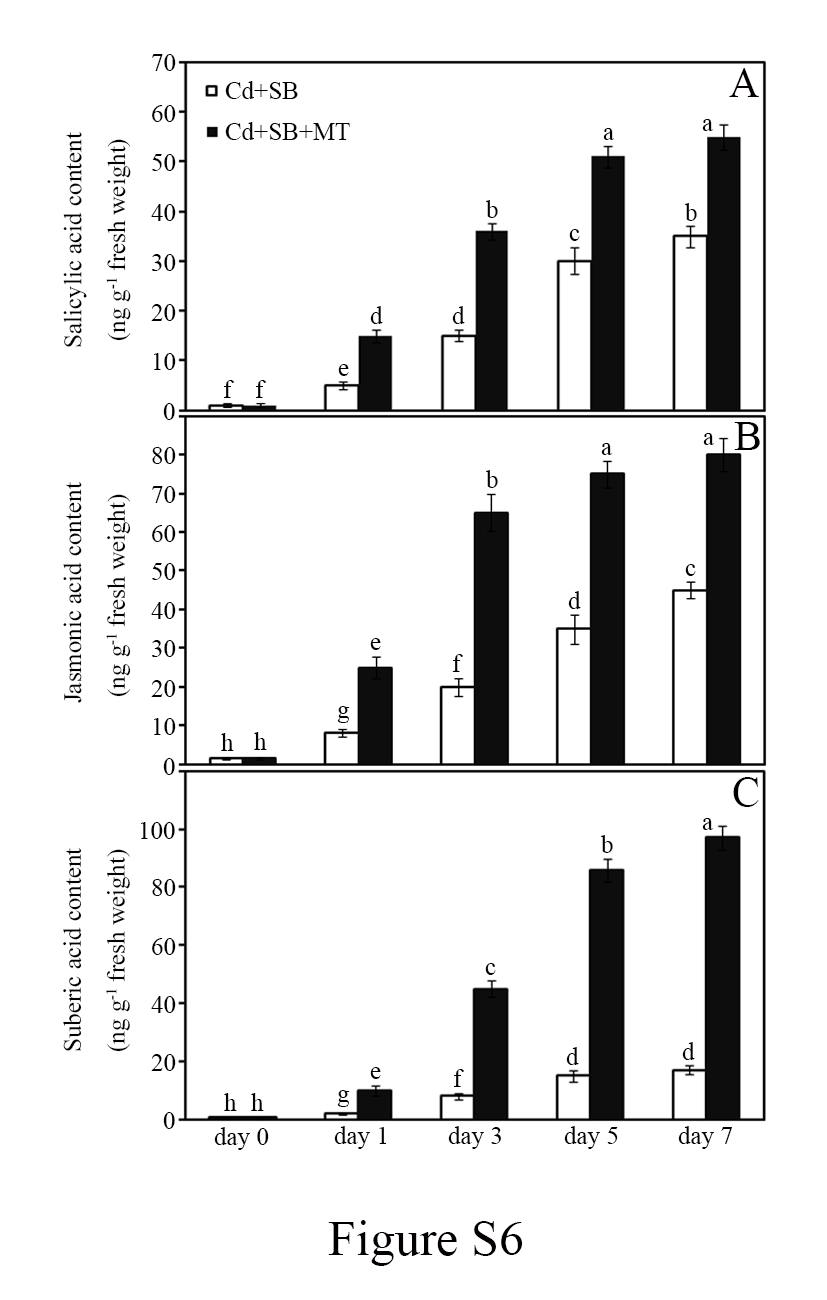

Supplement: Supplementary Table 1 — Dry weight, disease index, and cadmium content in peanut seedlings subjected to combined cadmium and southern blight infection stress under different melatonin concentrations (0, 0.01, 0.1, and 1.0 mM) after 7 days of treatment. Data are presented as mean ± SD (n = 5). Different letters indicate significant differences among treatments according to Duncan’s multiple range test (p < 0.05). [file DataSheet1.zip › Suppl Mater/Figure S6.tif]
