# Supplementary material for: Dual defense: melatonin simultaneously mitigates cadmium toxicity and southern blight in peanut
Source: Front Plant Sci. 2025 Oct 16;16:1686151. doi: 10.3389/fpls.2025.1686151 (PMC12571740; doi:10.3389/fpls.2025.1686151)
Supplement: Supplementary Table 1 — Dry weight, disease index, and cadmium content in peanut seedlings subjected to combined cadmium and southern blight infection stress under different melatonin concentrations (0, 0.01, 0.1, and 1.0 mM) after 7 days of treatment. Data are presented as mean ± SD (n = 5). Different letters indicate significant differences among treatments according to Duncan’s multiple range test (p < 0.05). [file DataSheet1.zip › Suppl Mater/Table S1 and 2.doc]

**Supplementary materials**
**Table S1 Dry weight, disease index, and cadmium content in peanut seedlings subjected to combined cadmium and southern blight infection stress under different melatonin concentrations (0, 0.01, 0.1, and 1.0 mM) after 7 days of treatment. Data are presented as mean ± SD (n = 5). Different letters indicate significant differences among treatments according to Duncan’s multiple range test (p < 0.05).**

|  | **0 mM** | **0.01 mM** | **0.1 mM** | **1 mM** |
| --- | --- | --- | --- | --- |
| **Dry weight (g seedling^-1^)** | 2.37 ± 0.03 | 2.39 ± 0.06 | 2.43 ± 0.07 | 2.15 ± 0.05 |
| **Disease index (%)** | 24.2 ± 2.3 | 19.3 ± 1.6 | 8.9 ± 1.2 | 12.5 ± 1.5 |
| **Cd content (mg kg^-1^ DW)** | 231.5 ± 8.4 | 205.8 ± 6.7 | 114.7 ± 11.3 | 145.7 ± 5.8 |

**Table S2** Primer sequences used for qRT-PCR analysis of JA biosynthesis and Cd transporter genes in this study.

| **Gene name** | **Primer name** | **Primer (5'-3')** |
| --- | --- | --- |
| ***AhLOX7*** | *AhLOX 7-F* | GGAGCCATCTTGGGGTGA |
|  | *AhLOX 7-R* | CCCTTTCCAGTGAGTCCAG |
| ***AhOPR3*** | *AhOPR3-F* | ACAAGCCCCTCAAACCAC |
|  | *AhOPR3-R* | TCAACCCCATCAAACCCT |
| ***ABCC 3*** | *ABCC3_F* | GTTGCCTTAGCCTTGTTG |
|  | *ABCC3_R* | GTTGCCTTCATCCTTGTATC |
| ***ABCC4*** | *ABCC4_F* | CACACTCCACACAAATCAA |
|  | *ABCC4_R* | TCGCCATTCAACAATAACC |
| ***β-actin*** | *β-actin_F* | CTGAAAGATTCCGATGCCCTGA |
|  | *β-actin_R* | AACCACCACTCAAGACAATGTTACCA |
